# Hepatocyte TM4SF5-mediated cytosolic NCOA3 stabilization and macropinocytosis support albumin uptake and bioenergetics for hepatocellular carcinoma progression

**DOI:** 10.1038/s12276-025-01438-9

**Published:** 2025-04-04

**Authors:** Haesong Lee, Ji Eon Kim, Eun-Ae Shin, Yangie Pinanga, Kyung-hee Pyo, Eun Hae Lee, Wonsik Kim, Soyeon Kim, Chang Sup Lim, Kyung Chul Yoon, Jung Weon Lee

**Affiliations:** 1 Department of Pharmacy, College of Pharmacy, Seoul National University, Seoul, Republic of Korea; 2 Research Institute of Pharmaceutical Sciences, College of Pharmacy, Seoul National University, Seoul, Republic of Korea; 3 Department of Surgery, Seoul National University Boramae Medical Center, Seoul, Republic of Korea

**Keywords:** Endocrine system and metabolic diseases, Liver cancer, Mechanisms of disease, Cancer metabolism

## Abstract

Transmembrane 4 L six family member 5 (TM4SF5) is involved in hepatocellular carcinoma (HCC) development and progression. Although TM4SF5 also promotes migration and invasion, it remains unclear how the metabolic context affects metastatic potential. Here we explored how TM4SF5 affects albumin uptake for HCC progression using TM4SF5 knockout or reintroduced hepatocyte and animal systems. Serum-deprived hepatocytes formed filopodia-like processes depending on TM4SF5 expression, which was altered by albumin replenishment for membranous PIP_3_-dependent macropinocytosis. Macropinocytosis required nuclear receptor coactivator 3 (NCOA3) stabilized in the cytosol and PTEN inactivation via binding to TM4SF5_WT_. TM4SF5-mediated albumin uptake led to ATP-linked respiration and cellular migration. Tumor tissues from liver-orthotopically xenografted mice fed a high protein diet or human liver cancer tissues showed TM4SF5-dependent macropinocytosis and NCOA3-correlated metastatic features, unlike mice fed a normal chow diet or human nontumor regions. These observations indicate that serum albumin availability to TM4SF5-positive HCC could support multifocality and intrahepatic metastasis, which may provide insights into clinical observations of multiple small tumor nodules surrounded by areas with high serum albumin levels.

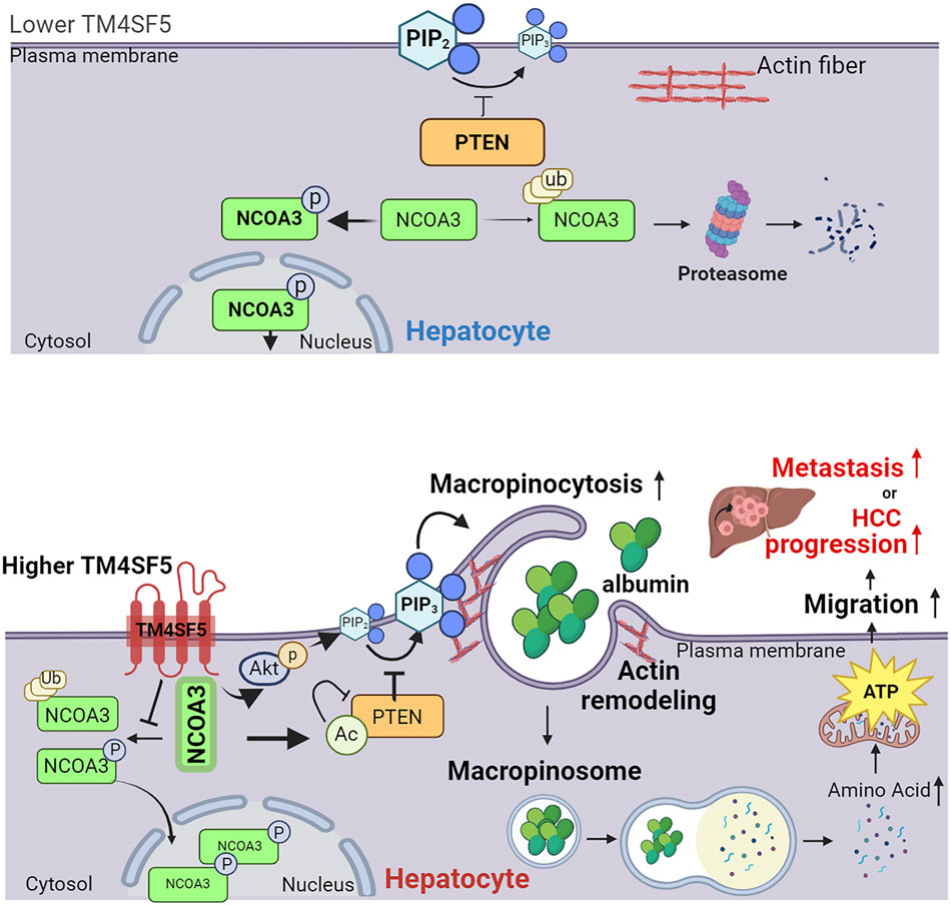

## TM4SF5 drives albumin uptake in liver cancer progression

Hepatocellular carcinoma (HCC) is a complex liver cancer with varying causes and outcomes. Researchers are exploring how certain proteins, such as albumin (ALB), affect HCC progression. This study investigates the role of the protein TM4SF5 in HCC. TM4SF5 is involved in cell processes and may help cancer cells take up ALB from their surroundings. Here various cell lines and animal models were used to study how TM4SF5 affects ALB uptake and cell behavior. The researchers found that TM4SF5 helps cancer cells absorb ALB through a process called macropinocytosis, which supports energy production and cell movement. This uptake is linked to changes in cell structure and increased energy production, aiding cancer spread. This study suggests that targeting TM4SF5 could slow HCC progression by disrupting ALB uptake. Future research could explore therapies that block TM4SF5 to prevent liver cancer growth and spread. This summary was initially drafted using artificial intelligence, then revised and fact-checked by the author.

## Introduction

Hepatocellular carcinoma (HCC) is a heterogeneous disease with diverse etiology, concomitant conditions, tumor biology and survival rates^1^. HCC progression is usually evaluated morphologically based on tumor diameter and number^2^. In addition, the identification of effective tumor markers to evaluate clinical outcomes of patients with HCC^3^ has previously indicated good predictive abilities of serum albumin (ALB), bilirubin, α-fetoprotein, the *Lens culinaris* agglutinin-reactive fraction of α-fetoprotein and des-γ-carboxy prothrombin^1,4^. Beyond diagnosis, ALB manipulation for therapeutic purposes has been intensively tested and adapted to treat liver diseases including cirrhosis^5^, although indications for nonspontaneous bacterial peritonitis are still controversial^6^.

As a major serum protein component, ALB is synthesized from hepatocytes, and a large portion of ALB is released into intravascular and interstitial spaces, where it plays multifunctional roles within homeostatic or pathological environments^5^. Although the biological mechanism and clinical importance of serum ALB for the diagnosis and prevention of liver cancers need to be further elucidated, many studies on the association between serum ALB level and cancer risk report differing results depending on the cancer type. A notable inverse linear relationship was reported between serum ALB level ([ALB]_serum_) and liver cancer risk^7^, and a low (<3.5 g/dl) [ALB]_serum_ predicts worse overall survival than a high (≥3.5 g/dl) [ALB]_serum_ in patients with HCC^3^. Meanwhile, the importance of serum ALB for the survival time of patients with liver cancer appears complicated^8^. The [ALB]_serum_ can affect the survival of patients with HCC because of nodule number changes^9^. With regards to nodule numbers, a low [ALB]_serum_ (that is, [ALB]_serum_ <3.5 g/dl) correlates with signficantly shorter survival; meanwhile, in cases with [ALB]_serum_ ≥3.5 g/dl, patients with more than two nodules showed a very short median survival time (8 months (95% confidence interval (CI) 6–12 months)), compared with that of patients with less than two nodules (17 months (95% CI 14–22 months)), indicating that a higher [ALB]_serum_ may affect the malignancy of patients with certain HCC statuses^9^. However, in patients with HCC with small tumors (<5 cm diameter), ALB levels ≥3.5 g/dl but not those <3.5 g/dl significantly correlate with more tumor nodules (more than three), which demonstrates the greater multifocality of small tumors within higher ALB-available environments and the greater multifocality of large tumors (≥5 cm diameter) within lower ALB-available environments^10^. However, it remains unclear how serum ALB mediates or modulates tumor number or size expansion at the molecular level.

Cancer cells uptake necrotic cell debris and recycle proteins from dying cells as nutrient sources when the nutrient supply is limited^11^. Unlike receptor-associated endocytosis, macropinocytosis is a metabolic route through which liver cancer cells uptake extracellular proteins or necrotic cell debris, leading to HCC growth under hypoxic conditions^12^. Differentially expressed macropinocytosis-related genes (DEMRGs)^13^, were correlatively identified from comparisons between normal liver tissue and HCC tumor samples from The Cancer Genome Atlas (TCGA). Macropinocytosis involves actin remodeling via EGFR–Pak signaling in pancreatic ductal adenocarcinoma tumors for ALB uptake^14^. As macropinocytosis involves the reuse of extracellular proteins or necrotic cell debris, it can be induced by PTEN deficiency and AMPK activation involving PIP_3_ accumulation and following energy stress, respectively; thus inhibition of macropinocytosis could be an effective strategy to target metabolism in late-stage prostate cancer, a tumor class known for its enigmatic nutrient dependency^15^. However, it is unclear how nutrient needs are linked to PTEN activity, especially in HCC. Furthermore, clinical benefits could be achieved by understanding how serum ALB may affect metabolic routes and activity in HCC development and progression.

Transmembrane 4 L six family member 5 (TM4SF5) is involved in HCC^16,17^ and colon cancer development^18,19^. It is similar to tetraspanins by virtue of its membrane topology, with four transmembrane domains, two (long or short) extracellular loops and cytosolic N- and C-terminal tails^20^ with N-glycosylation and palmitoylation^21^. TM4SF5 forms protein–protein complexes at different subcellular membranes with various receptors or proteins, such as EGFR, integrins^22^, cytosolic c-Src^23,24^ or glucose (GLU) transporters^25^. Therefore, TM4SF5 can form massive protein–protein complexes, presumably as a signaling hub of TM4SF5-enriched microdomains, similar to tetraspanin-enriched microdomains^26,27^. TM4SF5 is also involved in the regulation of focal adhesion turnover of COS7 kidney fibroblast-like cells^28^, hepatocyte morphology^29^ and crosstalks with integrins during migration and invasion^30^. In addition to focal adhesion relevant to cell–extracellular matrix (ECM) adhesion, TM4SF5 is located at cell–cell contacts (adhesions), resulting in efficient cell cycle progression and proliferation^31^. Therefore, hepatocyte TM4SF5 participates in the regulation of actin-based morphological changes during biochemical processes in a dynamic manner and in abnormal cell functions resulting in cellular malignancy. Cancer cells can change morphology, for example, during macropinocytosis to scavenge nutrients, leading to metabolic reprogramming^32^. Thus, it is worthwhile to examine whether hepatocyte TM4SF5 promotes macropinocytosis-mediated ALB uptake in nutrient-limited conditions during HCC development and/or progression.

Here, we investigated how hepatocyte TM4SF5 could modulate extracellular ALB uptake, leading to sufficient ATP synthesis for tumor progression. We hypothesized that TM4SF5 might promote ALB uptake and catabolism for ATP synthesis to support cellular migration during HCC development and progression. We observed that hepatocyte TM4SF5 caused cytosolic stabilization of NCOA3, which binds to TM4SF5 and PTEN, eventually resulting in PTEN inactivation and PIP_3_ accumulation for macropinocytosis upon ALB replenishment in serum-free media (SFM), leading to ALB uptake for bioenergetics during cellular migration. Therefore, TM4SF5 could be a promising target to prevent metabolic programs from using ALB support for HCC progression.

## Materials and methods

### Ethics

All animal procedures were performed in accordance with Seoul National University Laboratory Animal Maintenance Manual procedures and with institutional animal care and use committee approval from the Institute of Laboratory Animal Resources, Seoul National University (SNU-221024-4-1). Animal experiments were also performed in accordance with the Animal Research: Reporting of In Vivo Experiments guidelines. A human paraffin-embedded liver cancer tissue array with matched adjacent normal liver tissues (LV1505a) was obtained from US Biomax Inc.

### Cells

The Huh7, Hep3B, HepG2, SNU761 and SNU449 cell lines were purchased from the Korean Cell Line Bank (Seoul National University). Huh7, HepG2 and Hep3B cells endogenously expressing TM4SF5 were maintained in Dulbecco’s modified Eagle’s medium (SH30243.01, HyClone), and SNU449 cells were grown in RPMI-1640 (SH30027.01, HyClone) supplemented with 10% fetal bovine serum (FBS; F0600, GenDEPOT) and penicillin/streptomycin (CA005, GenDEPOT). Huh7, HepG2 and Hep3B cells at 60–70% confluence were transfected with pSpCas9(BB)-2A-Puro (PX459) V2.0 (plasmid 62988, Addgene) using polyethylenimine (408727, Sigma-Aldrich). The control gRNA targeting sequence for adeno-associated virus integration site 1 was 5′-GGGCCACTAGGGACAGGAT-3′, and the sequences for *TM4SF5* (exon 2) were 5′-TCCGGGGATTGCAGCCGTT-3′ (no. 1), 5′-ATTGCAGCCGTTCGGGCAG-3′ (no. 2) and 5′-GATTGCAGCCGTTCGGGCA-3′ (no. 3). After 24 h, cells were selected by culturing on media containing puromycin 2 μg/ml for 3 days. After puromycin-resistant cells were trypsinized and diluted, they were seeded on 96-well plates to produce single cell-derived clones. Each clone was cultured to appropriate confluence and genotyped by direct sequencing and western blotting for TM4SF5 expression. Cells were checked for mycoplasma every other month. Different cell variants or clones were collected, their identity was confirmed and they were used in studies to generalize our observations. Control, TM4SF5 wild-type (TM4SF5_WT_) or TM4SF5_A132V_ plasmids were reconstituted and introduced into Huh7 knockout (Huh7_KO_) or Hep3B_KO_ via transfection or viral infection. Cell variant subscript numbers in the form of _X#Y_ depict KO cloning trial number X and clone number Y. TM4SF5 suppression was achieved by transfection of short interfering RNA against nonspecific sequences (siNS) or human *TM4SF5* (Table 1).

**Table 1:** Sequences for quantitative PCR with reverse transcription primers or siRNA targets.

Gene names	Sequences	
	Forward primer (5′ → 3′)	Reverse primer (5′ → 3′)
ITGA4	TGAATGTGTCCTTGTTTAATGCTG	TTGTACCACGCCAGAGTTATC
ITGA5	ATACTCTGTGGCTGTTGGTG	CTGTTCCCCTGAGAAGTTGTAG
DMD (Dystrophin)	GAACTGGCTGCTGAATGTTTATG	CGCTTCGATCTCTGGCTTATT
SLC1A5	GAAGTGCGTGGAGGAGAATAA	CAGCCAGGATCAAGGAGATATG
GAPDH	CCAGCCGAGCCACATCGCTC	ATGAGCCCCAGCCTTCTCCAT
Gene names	siRNA target sequence	
	Forward (5′ → 3′)	Reverse (5′ → 3′)
ITGA2	GAAACGCCCUUGAUACUAAAAAUTT	AAAUUUUUAGUAUCAAGGGCGUUUCUG
NCOA3	AAACCAGCAGAAUAUCAUGAUUUCT	AGAAAUCAUGAUAUUCUGCUGGUUUGG
ITGA4	GGUACUGCAUUCUGAAUCAGAAUCT	AGAUUCUGAUUCAGAAUGCAGUACCUG
ITGA5	CUACAAGCUUGGAUUCUUCAAACGC	GCGUUUGAAGAAUCCAAGCU UGUAGAG
DMD (Dystrophin)	CUUAGUAUCAGUCAUGACAGAUGAA	UUCAUCUGUCAUGACUGAUACUAAGGA
SLC1A5	GAUCUUGCGAGAAAUAUCUUCCCTT	AAGGGAAGAUAUUUCUCGCAAGAUCC
siTM4SF5_#4_	CCA UCU CAG CUU GCA AGU C	GAC UUG CAA GCU GAG AUG G
siTM4SF5_#7_	CCUCCU GCU GGU ACC UAA U	AUU AGG UAC CAG CAG GAG G
siNS	GenePharma negative control no. A06001	

Additionally, SNU761 or SNU449 cells lacking endogenous TM4SF5 expression were also produced for stable cell lines following transfection or transduction of empty, TM4SF5 WT or A132V mutant expression vectors or retroviral particles^21^. TM4SF5 expression was confirmed using a rabbit anti-TM4SF5 polyclonal antibody^33^.

### Western blots

Cells were cultured in normal 10% FBS-containing media or replated on culture wares precoated with poly-lysine (P4832, Sigma) or different extracellular matrix (10 μg/ml collagen type I (5056, Advanced Biomatrix), fibronectin (CLS356008, Merck) or laminins (354232, Life Sciences)). Cells were incubated in serum-free Dulbecco’s modified Eagle’s medium or RPMI-1640 media (SFM with basal GLU containing or without GLU) for 3 h. Cells then did or did not undergo replenishment with GLU (10 or 25 mM for SNU449, HepG2 or Huh7 cells, respectively; G8270, Sigma), ALB (3.6 mg/ml; A8806, Sigma) or GLU + ALB for 15 h or the indicated times, via each addition into (basal) GLU-free SFM. Cells in normal culture or as treated as described above were collected for whole-cell lysates using modified RIPA lysis buffer (50 mM Tris–HCl, pH 7.4, 150 mM NaCl, 0.5% sodium deoxycholate, 0.1% SDS and 1% Triton ×100). Lysates were normalized before immunoblot analysis using the following primary antibodies: β-actin (sc-47778), pS^473^AKT1 (sc-7985-R) and AKT1 (sc-8312) from Santa Cruz; HA (3724), pS^2448^-mTOR (5536), mTOR (2983s), pT^172^AMPK (2535s), AMPKα (5832s), pS^63^-JUN (9281s), c-JUN (9165s), p-ERK1/2 (9102s), ERK1/2 (9101s), α-tubulin (2125), NCOA3 (5765), PTEN (9559s), pT^24^NCOA3 (5765), ubiquitin (S43124) or acetyl-lysine (9441s) from Cell Signaling Technology; strep-HRP (2-1509-001) from IBA Lifesciences; ALB (213-MSM4-P1) from NeoBiotechnologies; or purified anti HA-11 epitope tag antibody (901501) from Biolegend. An anti-TM4SF5 antibody was generated by immunizing rabbits with a TM4SF5 C-terminal or long extracellular loop two-sequence peptide^33^. In some cases, the ratio values of band intensities of certain immunoblots measured by ImageJ software were normalized to those of a loading control or the total form of the molecule was presented.

### Immunoprecipitation or pulldown

Cells were also lysed in Triton X-100 lysis buffer (40 mM HEPES, pH 7.4, 150 mM NaCl, 1 mM EDTA and 0.5% Triton X-100) with protease inhibitors (P3100-005, GenDEPOT). Whole-cell lysates were incubated with streptavidin agarose (20353, Thermo Fisher Scientific) or protein A-agarose beads (P9201-100, GenDEPOT) at 4 °C for 4 h. Beads were washed with ice-cold lysis buffer once and wash buffer (40 mM HEPES, pH 7.4, 500 mM NaCl, 1 mM EDTA and 0.5% Triton X-100) twice, followed by a wash with ice-cold PBS. Washed beads were eluted in 2× SDS–PAGE sample buffer and boiled for 5 min before immunoblot analysis. Ratio values of band intensities of certain immunoblots measured by ImageJ software were normalized to those of a loading control or the pulled-down forms of the molecule were presented.

### Immunofluorescence

A cover glass was precoated with 10 μg/ml fibronectin, laminins, collagen I or poly-l-lysine in PBS at room temperature (RT) for 1 h and briefly rinsed three times with PBS. Twenty thousand cells were replated on each precoated cover glass in 12-well plates for 15 h. Cells were transfected with indicated cDNA constructs or siRNAs (Table 1) using Lipofectamine 3000 (L3000015) or RNAiMAX (2529659, Invitrogen) for 24 h. Cells were then manipulated for serum starvation and nutrient repletion as described above for 15 h. Cells were treated with control compound (4′-methoxy-4-dihydroxychalcone), specific TM4SF5 inhibitor 4′-(*p*-toluenesulfonylamido)-4-hydroxychalcone (TSAHC; 2 or 5 μM in DMSO^29^), 5-(*N*-ethyl-*N*-isopropyl) (EIPA, 10 μM) or bpV (Phen, 1 μM). The cover glass was then fixed with 3.7% formaldehyde for 20 min, permeabilized with 0.3% Triton X-100 in PBS for 10 min, and blocked with 5% bovine serum albumin (BSA) for 1 h at RT. Cells were imaged for mCherry or GFP, stained with rhodamine–phalloidin (R415, Invitrogen) or 4,6-diamidino-2-phenylindole (DAPI; D9542, Sigma), or incubated with primary antibodies diluted in 1% BSA in PBS for 15 h at 4 °C. The primary antibodies were anti-NCOA3 (5765) or anti-pT^24^NCOA3 (2979) from Cell Signaling Technology. The cover glass was then washed three times with PBS and incubated with donkey anti-rabbit IgG (H + L) highly cross-adsorbed secondary antibodies (1:500, Alexa Fluor 488 (goat: A11055 and mouse: A21202)) or donkey anti-mouse IgG (H + L) highly cross-adsorbed secondary antibody (1;500, Alexa Fluor 555 (goat: A21432 and mouse: A31570)) from Invitrogen in PBS at RT for 1 h. The cover glass was then washed three times with PBS and mounted on slide glasses using ProLong Gold Antifade (P36930, Invitrogen). Cells were randomly visualized using a Nikon Eclipse Ti microscope with a C2 confocal system and normal Photomultiplier tube (PMT, Nikon) and CFI Apochromat Lambda S 40× or CFI Apochromat Lambda S 60× NA1.49 oil immersion objective (Nikon) after excitation with 405, 488 and 561 nm lasers. Images were analyzed using IMARIS (Oxford Instruments) or NIS-Elements software (Nikon).

### Live imaging

The methods are described in the Supplementary information.

### Microscopic quantification of filopodia-like processes

Actin filaments of cells were stained with rhodamine–phalloidin (R415, Invitrogen). Images were collected at RT using a C2+ confocal microscope (Nikon). The number of filopodia-like processes was counted using the Fiji plugin ADAPT (v1.193) as previously described^34^. Using the ADAPT plugin, each image with a cell body was segmented from the background image. To isolate only filopodia-like structures from the periphery of a cell, a segmented cell image was eroded. After removing the eroded cell image from the segmented boundary image, filopodia-like structures were counted by specifying a maximal filopodia size of 20 μm^2^ and minimal size of 1 μm^2^ (ref. ^35^). The microscopic analyses were performed at least three times independently for the same experimental conditions, and then the quantifications of the image stains from multiple cells in each experiment were performed with combined data points from the experiment trials. One dot datum in the graphs depicts the number of protrusions per cell (that is, all around a cell over edges), although the microscopic images around cell edges show a part of the edge for figure size limitations.

### In vitro macropinocytosis/FITC-ALB uptake assay

Eight-well chamber slides were precoated with collagen I and washed three times with PBS before cell seeding. Cells were reseeded in the chamber wells and subjected to serum or GLU starvation for 3 h before GLU-free SFM was or was not replenished with GLU alone, ALB alone or GLU + ALB for 15 h. During nutrient replenishment, fluorescein isothiocyanate (FITC)-tagged ALB (FITC-ALB, 0.01 mg/ml; A13100, Thermo Fisher Scientific) was administered before counting macropinosomes (confocally visualized with FITC-ALB inside of a cell) from multiple cells^36^ using IMARIS software (Oxford Instruments). The macropinosome index was calculated as the number of macropinosomes per cell. The FITC-ALB uptake analyses were performed three times independently for same experimental conditions, and the quantification data points from mutliple cells of experimental trials were all collected and combined for comparisons.

### Cell tracking for cellular migration

Cells were manipulated as described in ‘Microscopic quantification of filopodia-like processes’ or ‘In vitro macropinocytosis/FITC-ALB uptake assay’ sections, with regards to extracellular nutrient conditions or replenishments. Time-lapse images were collected with an IX81-ZDC microscope (Olympus) equipped with a UPLSAPO 10× 2, NA0.4 Super Apochromatic objective (Olympus) via a controller (CU-109; Live Cell Instrument) every 20 min in a chamber maintained at 37 °C and 5% CO_2_ at 40–60 ml/min for 16 h. All images were captured with a Prime sCMOS camera (4.2 megapixel, BAE CIS-2020F sCMOS; Photometrics) and analyzed with MetaMorph software (Molecular Devices LLC). Each cell was tracked using the ‘Track object’ application and processed to calculate the average speed of each cellular migration^21^.

### APEX2 staining and transmission electron microscopy for the analysis of protein binding to TM4SF5 or ALB

The methods are described in the Supplementary information.

### Mice and in vivo macropinocytosis assay

Six-week-old BALB/cAnN-nude male mice (Orient Bio) were used for orthotopic implantation of cells expressing TM4SF5-negative (SNU449_Cp_-luciferase) or TM4SF5-positive (SNU449_T7_-luciferase) reporter cells (1 × 10^6^ cells/mouse) in Matrigel (354230, BD Biosciences)^37^. Mice were housed under a dark/light cycle of 12 h, ambient temperature of 22 °C and humidity of 30–70%. Mice were given ad libitum access to a normal chow diet (NCD; 20% protein) or a high GLU (20% w/v with filtered water) and protein (40% protein) diet (HGProD; TD 90018, Envigo). Mice were subcutaneously injected with 100 mg/kg d-luciferin (88293, Thermo Fisher Scientific), and bioluminescent imaging was repeatedly performed using a PE-IVIS Spectrum In Vivo Imaging System (IVIS) (PerkinElmer). On day 20 after cell injection, mice were intratumorally injected with FITC-ALB (0.2 mg/mouse). After 90 min, mice were killed without pain, and their livers were imaged for in vivo macropinocytosis-mediated FITC-ALB uptake^38,39^. Tumor tissues after in vivo imaging were collected and diced into 3 mm^3^ pieces immediately after euthanasia, washed with PBS and fixed in 3.7% formaldehyde at 4 °C overnight. Fixed tumor tissue was stained with DAPI for 30 min. Images were captured using a confocal laser microscope with a Nikon Plan-Apochromat 20× objective to visualize the green signal for FITC-ALB.

### Immunohistochemistry

The methods are described in the Supplementary information. Hepatomas and matched adjacent normal liver tissues were obtained from Professor Surh YJ (Seoul National University), which were reported in a previous report^40^ that was performed in accordance with the Declaration of Helsinki of the World Medical Association and approved by the Institutional Review Board of Gachon University Gil Medical Center (authorization number: GDIRB2022-127).

### Seahorse metabolic analysis

To measure the extracellular acidification rate (ECAR) and oxygen consumption rate (OCR) of cells, a Seahorse XFe24 analyzer (Agilent) was used. Cells were seeded on XF24 culture plates precoated with collagen I (10 μg/ml) at 2.5 × 10^4^ cells/well. Three hours later, cells were starved from serum and/or GLU and treated with EIPA (10 μM; A8806, Sigma), chloroquine (25 μM; C6628, Sigma) or bafilomycin A1 (50 nM; B1793-2UG, Sigma) for 15 h. Cells were washed and incubated in Seahorse Assay Medium supplemented with 1 mM pyruvate, 2 mM glutamine, 25 mM GLU and/or ALB (3.6 mg/ml) in a 37 °C non-CO_2_ incubator for 45 min. General seahorse OCR or ECAR analyses were then performed according to the manufacturer’s protocols, with assay systems including the following compounds depending on the experiment: oligomycin A (2 μM), FCCP (3 μM), rotenone/antimycin (0.5 μM), 2-DG (250 mM), ALB (3.6 mg/ml), GLU (optimized concentration: 0.10–5.0 mM), DMSO, EIPA (10 μM), chloroquine (25 μM) or bafilomycin A1 (50 nM). In some experiments, free amino acid was administered at different concentrations before the seahorse OCR measurement based on a previous report on the amino acid composition of BSA^41,42^: Asp (0.43 mg/ml), Ser (0.15 mg/ml), glutamic acid (0.63 mg/ml), Gly (0.14 mg/ml), His (0.14 mg/ml), Arg (0.20 mg/ml), Thr (0.18 g/ml), Ala (0.18 mg/ml), Pro (0.16 mg/ml), Tyr (0.18 mg/ml), Val (0.19 mg/ml), Lys (0.37 mg/ml), Ile (0.10 mg/ml), Leu (0.35 mg/ml) and Phe (0.22 mg/ml). In some experiments, Phe was administered together with EIPA before OCR measurement. The area under the curve for glycolytic or respiratory activity was calculated and presented as graphs using Seahorse Wave Desktop software.

### Live three-dimensional holotomography and PTEN phosphatase assay

The methods are described in the Supplementary information.

### Public data analysis

Gene Set Enrichment Analysis or Gene Ontology (GO) enrichment analyses were performed using RNA-sequencing data (SRA accession number: PRJNA770813; RNA-seq using stably TM4SF5-transfected SNU449 cells over TM4SF5-null SNU449 cells) and gene sets from Kyoto Encyclopedia of Genes and Genomes (ftp.broadinstitute.org://pub/gsea/gene_sets/c2.cp.kegg.v2023.1.Hs.symbols.gmt) with GSEA_4.1.0. STRING functional enrichment test^43^ was done using the TM4SF5-binding protein lists analyzed by a liquid chromatography–tandem mass spectrometry approach of TM4SF5 precipitates^21^. Co-expression graphs and heat maps were drawn using the TCGA–liver hepatocellular carcinoma (LIHC) dataset at Cbioportal (https://www.cbioportal.org/). In addition, the probability of survival ratio was calculated using TCGA–LIHC dataset including transcripts levels and patient information including [ALB]_serum_ to see the importance of [ALB]_serum_ in the relationship between *TM4SF5* and *NCOA3* levels for poor survival ratios. TM4SF5 expression graphs for nontumor and tumor tissue (from the TCGA–LIHC dataset supplemented with GTXe projects^44^) were obtained from GEPIA2-Expression DIY-Box. In addition, other cancer types including kidney renal clear cell carcinoma (KIRC), colon adenocarcinoma (COAD) and stomach adenocarcinoma (STAD) with an increase TM4SF5 in tumor samples over normal-like counterparts and breast invasive carcinoma (BRCA), skin cutaneous melanoma (SKCM), adrenocortical carcinoma (ACC) and uterine corpus endometrial carcinoma (UCEC) with non-altered TM4SF5 expression between tumor and normal-like samples, were also analyzed for *TM4SF5* and related gene mRNA levels.

### Statistical analysis

Statistical analyses were performed using Prism software version 9.0 (GraphPad). Two-way analysis of variance (ANOVA), one-way ANOVA, Mann–Whitney *U* tests or unpaired Student’s *t*-tests were performed to determine statistical significance. A value of *P* < 0.05 was considered statistically significant. The symbols *, **, *** or **** indicate statistical significance of *P* < 0.05, 0.01, 0.001 or 0.0001, respectively. Additionally, ns indicates nonsignificance.

## Results

### Hepatocyte TM4SF5 causes differential filopodia-like process formation depending on extarcellularly available nutrients

**Fig. 1:**
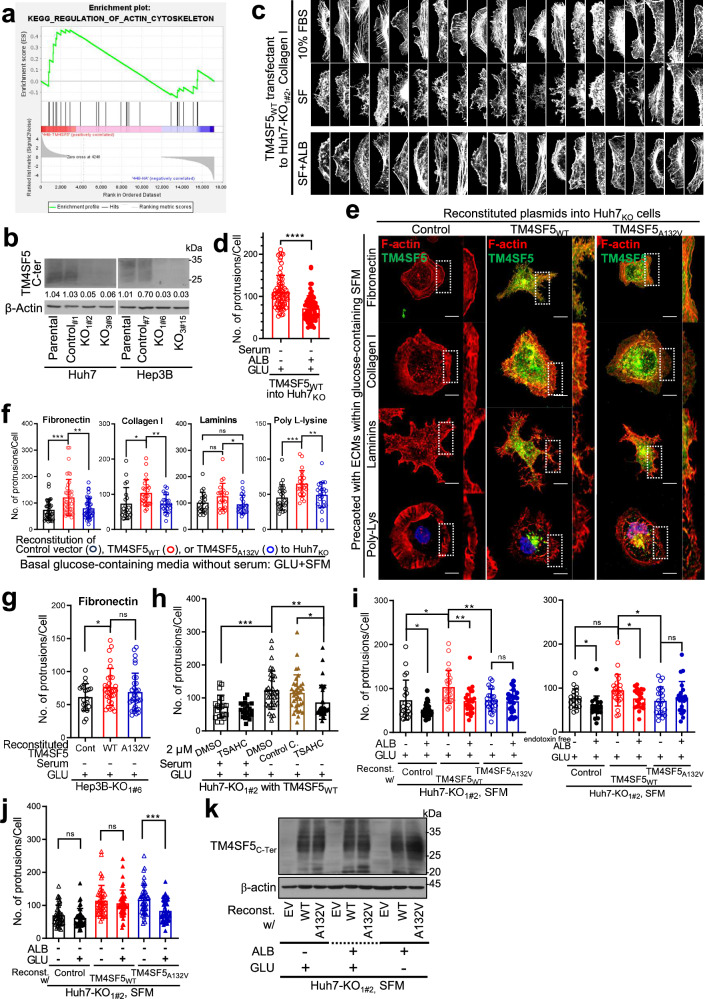
Hepatocyte TM4SF5 has differential effects on filopodia-like processes depending on available nutrients. **a** Enrichment plot by the Kyoto Encyclopedia of Genes and Genomes pathway of actin cytoskeleton organization depending on ectopic TM4SF5 expression in SNU449 hepatocytes. **b** TM4SF5 immunoblots of representative clones of TM4SF5-KO or control (non-KO) hepatocyte variants via a CRISPR–Cas9 approach from parental Huh7 and Hep3B cells with endogenous TM4SF5. The subscripts indicate different Huh7_KO_ cell variants. The band intensity ratio values were measured by ImageJ and normalized to those of the loading control. **c**, **d** TM4SF5_WT_-HA cDNA transfected into Huh7-KO_1#2_ cells for 24 h were replated on collagen I (10 μg/ml) without (SFM) or with 10% FBS or SFM + ALB (3.6 mg/ml) with basal GLU before phalloidin staining (**c**) or onto collagen I in basal GLU-containing SFM with or without ALB (**d**). In **c**, representative images with pseudo-colors per HA tag-stained cell are presented (*n* ≥ 20, each data point is a value from one individual cell). In **d**, the automatic quantification of filopodia-like processes (or protrusions) per cell visualized by rhodamine–phalloidin staining was performed using the Fiji plugin ADAPT (v1.193). Each dot indicates the protrusion numbers of a cell. **e**–**g** Huh7-KO_1#2_ (**e** and **f**) or Hep3B-KO_1#6_ (**g**) cells reconstituted with the indicated cDNA were replated to different ECM or poly-l-lysine (10 μg/ml) for 16 h in the GLU + SFM condition before fluorescence staining of the HA tag: rcn (**e**) and quantified for filopodia-like protrusions (**f** and **g**). **h**–**j** Huh7_KO_ cell variants on collagen I were treated with DMSO, control compound or TSAHC as above (**h**), with replenishment of GLU alone or GLU + ALB to SFM (**i**) or of none or GLU alone (**j**), before imaging and protrusion quantification. *P* values were calculated via one-way ANOVA or unpaired Student’s *t*-tests, and *P* < 0.05 was considered statistically significant. **k** Huh7-KO_1**#**2_ cells transduced with lentivirus for different cDNAs were manipulated as in **c**, before immunoblots. Data represent three independent experiments. See also Supplementary Figs. 1, 2 and 4.

TM4SF5 is shown to be involved in cell–cell adhesion^45^, cell–ECM adhesion^28^ and cellular morphology changes^16^. Furthermore, an RNA-sequencing dataset (SRA accession number: PRJNA770813) from TM4SF5-negative and TM4SF5-positive SNU449 cells showed enrichment of diverse actin organization-related genes in TM4SF5-positive cells (Fig. 1a). In addition, GO enrichment analysis of the RNA-sequencing dataset revealed enrichment of GO terms related to actin dynamic and cell migration in TM4SF5-expressing hepatocytes, compared with those in TM4SF5-lacking hepatocytes (Supplementary Fig. 1a). Furthermore, among the TM4SF5-binding proteins determined via TM4SF5-strep pulldown proteomic analysis using liquid chromatography–tandem mass spectrometry approaches^21^, STRING functional enrichment analysis showed relevance to actin organization and cell motility (Supplementary Fig. 1b). Thus, TM4SF5 expression might be related to dynamic morphological changes and extracellular conditions (for example, the absence or presence of nutrients and/or serum components) may further affect the TM4SF5-mediated effects, presumably leading to alterations in cell functions. To elucidate the roles of TM4SF5-mediated actin dynamics in metabolic activity in hepatocytes endogenously expressing TM4SF5, we first knocked out the gene using a CRISPR–Cas9 approach. Many different clones of control (Huh7-Cont_#1_ and Hep3B-Cont_#7_) and KO (Huh7-KO_1#2_, Huh7-KO_3#9_, Hep3B-KO_1#6_ and Hep3B-KO_3#15_) cells with diverse hepatocyte backgrounds (Fig. 1b) were obtained, before reconstitution with TM4SF5 WT or A132V mutant vectors. We manipulated different extracellular cues and then Huh7_KO_ cell variants were stained for actin after reintroduction of control (Huh7_KO_-control (that is, TM4SF5-lacking control Huh7-KO_1#2_)), TM4SF5_WT_ (Huh7_KO_-TM4SF5_WT_) or TM4SF5_A132V_ (Huh7_KO_-TM4SF5_A132V_) expression vectors into Huh7_KO_ (that is, Huh7-KO_1#2_) cells. Cells in GLU-containing SFM (that is, that still contain basal glucose) prominently formed filipodia-like or protrusive processes, which were abolished by ALB replenishment (Fig. 1c). By contrast, the filopodia-like morphology of TM4SF5_WT_ cells in GLU-containing SFM was reduced by replenishment with 10% FBS, but not by fatty acid (palmitic and oleic acid) or amino acid treatment (Supplementary Fig. 2a). Furthermore, FBS dialyzed at a 3 kDa molecular cutoff could reduce the processes on boundary edges of WT (Huh7_KO_-TM4SF5_WT_) cells in SFM (Supplementary Fig. 2a), indicating serum components smaller than 3 kDa, such as fatty acids and amino acids, did not effectively reduce boundary processes in TM4SF5_WT_ cells in SFM.

As TM4SF5-mediated effects depended on the SFM condition, we hypothesized that serum components (for example, ALB and glucose) might be critical for these effects. As ALB is the major protein in serum^46^, it was added at a concentration of 0.36 g/dl (3.6 mg/ml), although normal serum ALB in humans can range from 3.5 to 7.0 g/dl. Imaging software-based quantification of the protrusive processes of each (Huh7_KO_-)TM4SF5_WT_ cell upon serum deprivation without or with ALB replenishment (GLU-containing culture media) showed that the processes were notably reduced upon ALB treatment (Fig. 1d). Microscopic analyses were independently performed at least three times for each experimental condition, and staining quantifications from random images of mutliple cells in each experiment were combined. Interestingly, (Huh7_KO_-)TM4SF5_WT_ cells showed serum- and ALB-dependent changes in process formation patterns under different ECM-precoated conditions, including a poly-l-lysine-coated condition (Fig. 1e, f). In addition to TM4SF5_WT_, we evaluated the effects in Huh7_KO_ cells with reconstitution of the TM4SF5_A132v_ mutant, which was identified as a somatic mutation in Japanese patients with HCC (COSMIC sample ID: COSS2120737 and COSS2120738) that we found to cause a more elongated morphology than the WT (data not shown). Unexpectedly, the poly-l-lysine-coated condition produced similar results, which might suggest that TM4SF5-mediated effects on poly-l-lysine are supported by ECM components secreted by cells during overnight incubation of ~15 h (Fig. 1f). As we pursued more general aspects of hepatocyte TM4SF5-mediated effects, we occasionally used different hepatocyte cell lines and variants during this study. Thus, another hepatocyte Hep3B_KO_ cell line with the reintroduction of control, WT or A132V mutant vectors was replated on fibronectin to generalize the TM4SF5-mediated effects. Compared with Hep3B_KO1#6_ cells, TM4SF5-reintroduced cells showed increased protrusions in SFM-containing basal GLU (GLU + SFM; Fig. 1g), similar to the Huh7_KO_-derived cell lines. The serum deprivation-mediated increase in Huh7-TM4SF5_WT_ cell protrusions was further abolished by treatment with a specific TM4SF5 inhibitor, TSAHC^29^, but not by a control compound (Fig. 1h). Interestingly, TM4SF5_WT_ cells showed a significant decrease in the number of protrusions per cell upon ALB replenishment, whereas control Huh7_KO_ or Huh7_KO_-TM4SF5_A132V_ cells showed a reduction or no change in protrusions relative to the basal level, respectively (Fig. 1i, left). Furthermore, endotoxin/fatty acid-free ALB treatment produced consistent trends; protrusions in TM4SF_WT_ cells appeared to be increased compared with those in control (*P* = 0.0507) or TM4SF5_A132v_ cells (Fig. 1i, right). Depletion of basal GLU in SFM (that is, GLU-free SFM) resulted in increased protrusive processes in TM4SF5_A132V_ cells but no change in TM4SF5_WT_ cells (Fig. 1j and Supplementary Fig. 2b). When the SFM contained no (−) or 25 mM (+) GLU without ALB replenishment, control and TM4SF5_WT_ cells did not respond to GLU, although TM4SF5_WT_ and TM4SF5_A132V_ cells showed a larger number of protrusions per cell, higher than that of control cells in GLU-free SFM. TM4SF5_A132V_ cells showed a significant decrease in protrusion number upon GLU treatment, but TM4SF5_WT_ cells did not (Fig. 1j). Such changes in the number of protrusions per cell depending on extracellular serum, GLU and/or ALB levels were validated in another cell clone (Huh7_KO3#9_) with TM4SF5 reintroduction (Supplementary Fig. 2c). However, replenishment of nutrients in SFM did not alter TM4SF5 expression (Fig. 1k). Therefore, TM4SF5-positive hepatocytes showed dynamic morphological changes depending on extracellular serum components.

### Nutrient-dependent morphological alterations lead to ALB uptake

**Fig. 2:**
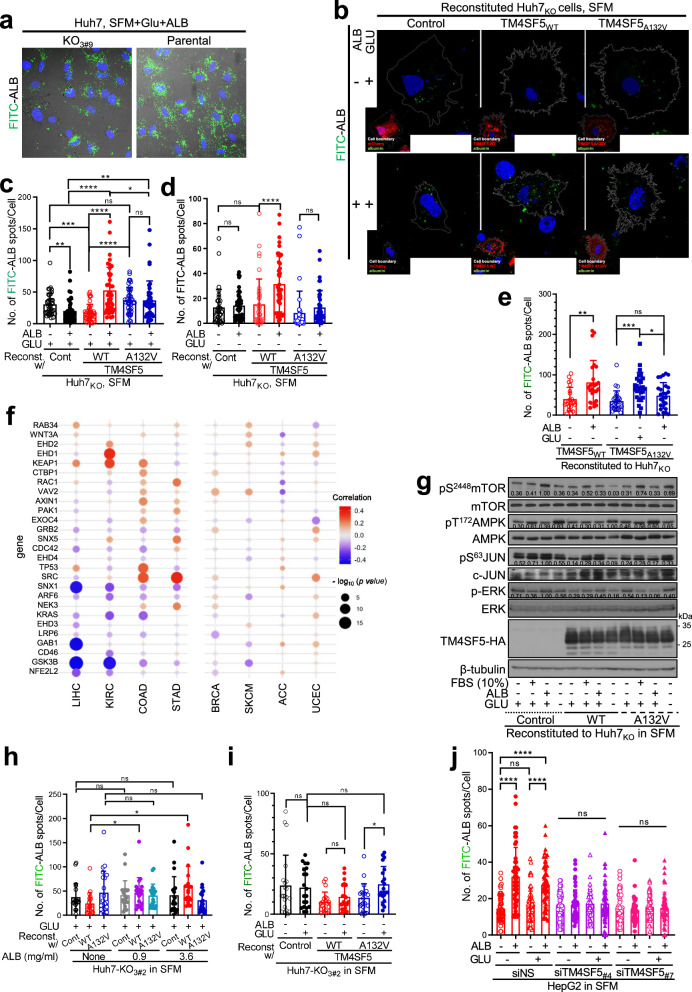
Nutrient-dependent morphological alterations led to ALB uptake. **a** Parental or *TM4SF5*-KO Huh7 cells replated on collagen I with ALB + GLU (3.6 mg/ml and 25 mM, respectively) replenishment to SFM were incubated with FITC-ALB (0.1 mg/ml) for 16 h, before imaging of FITC-ALB. **b**–**d** Huh7-KO_1#2_ cells reconstituted with pmCherry control, pmCherry-TM4SF5_WT_ or pmCherry-TM4SF5_A132V_ for 48 h were processed for analysis of macropinocytosis-mediated FITC-ALB uptake in replenishment of GLU alone (**b**) or ALB + GLU to SFM (**c**) or GLU-free SFM (**d**) for 16 h: representative images are shown (**b**) and the FITC-ALB spot numbers per cell counted using IMARIS software were graphed as mean ± s.e.m. (**c** and **d**). Each data dot depicts a value from one individual cell (**c** and **d**). **e** Huh7_KO_ cell variants prepared as in **b** were replated on collagen I and processed to analyze FITC-ALB uptake with or without replenishment of GLU or ALB alone to SFM for 16 h. **f** Bubble blots showing the fold changes in DEMRGs^13^ from TCGA datasets of cancer types with or without substantially altered *TM4SF5* expression in tumor tissue compared with nontumor tissues. **g** Huh7_KO_ cell variants reconstituted with control, TM4SF5_WT_-HA or TM4SF5_A132v_-HA cDNA were replated on collagen I with or without replenishment of GLU alone, GLU + FBS (10%) or GLU + ALB to SFM for 16 h before the collection of whole-cell extracts for immunoblots. The band intensity ratio values of a molecule were measured by ImageJ and normalized to those of a loading control or the total form of the molecule. **h**–**j** Huh7-KO_3#2_ cells (**h** and **i**) were reconstituted with TM4SF5_WT_ or TM4SF5_A132V_ or HepG2 cells transfected with control siRNA or TM4SF5 siRNA (no. 4 or 7 of the target sequence, **j** and Table 1) were then replated on collagen I with replenishment of ALB alone (0.9 or 3.6 mg/ml, **h**), GLU alone (**i**), or ALB + GLU (**j**) to SFM for 16 h before analysis of macropinocytosis-mediated FITC-ALB uptake. *P* values were calculated via one-way ANOVA or unpaired Student’s *t*-tests, and *P* < 0.05 was considered statistically significant. Data represent three independent experiments. See also Supplementary Figs. 3 and 4.

We next investigated whether hepatocytes could uptake ALB in a TM4SF5-dependent manner. Therefore, we administered FITC-ALB (0.1 mg/ml for 16 h) to Huh7 parental (endogenously TM4SF5 expressing) and Huh7_KO_ cells in GLU-containing SFM (GLU + SFM) before confocal microscopic imaging of the intracellular FITC-ALB signal. TM4SF5-expressing parental Huh7 cells showed a greater intracellular FITC-ALB signal than Huh7_KO3#9_ cells (Fig. 2a). When FITC-ALB uptake into hepatocytes was analyzed for 16 h using cells not expressing or expressing TM4SF5 forms, ALB (3.6 mg/ml) treatment of cells in 25 mM GLU + SFM significantly enhanced FITC-ALB uptake in TM4SF5_WT_ cells, but reduced or did not change FITC-ALB uptake in control or TM4SF5_A132V_ cells (Fig. 2b,c). Even in the absence of GLU in the SFM (that is, GLU-free SFM), FITC-ALB uptake in TM4SF5_WT_ cells was substantially increased upon replenishment of ALB alone to SFM (that is, ALB + SFM), but that in control or TM4SF5_A132V_ cells was not increased (Fig. 2d). FITC-ALB uptake analyses were independently performed three times for the same experimental conditions, and quantification data from mutliple cells of experimental trials were combined for comparisons. Furthermore, TM4SF5_WT_ cells showed significantly increased uptake with ALB alone, whereas TM4SF5_A132V_ cells showed a significant increase in uptake with GLU alone, but not with ALB alone (*P* = 0.0976 for nonsignificance) (Fig. 2e). Interestingly, TM4SF5_WT_ cells showed an ALB-dependent increase in FITC-ALB uptake (independent of GLU levels), whereas TM4SF5_A132v_ cells showed a GLU-dependent increase without an additional increase with ALB replenishment (Fig. 2c,d). As FITC-ALB uptake can occur via macropinocytosis mediated by actin remodeling and receptor-mediated endocytosis^14^, we determined whether 27 DEMRGs identified in HCC TCGA datasets^13^ are linked to TM4SF5 overexpression in hepatic cancer cells. Among different cancer types in TCGA datasets and GTXe projects^44^, TM4SF5 was overexpressed in LIHC, KIRC, COAD and STAD, but was not altered or not expressed in other cancer types including BRCA, SKCM, ACC and UCEC (Supplementary Fig. 3). Interestingly, among the 27 DEMRGs^13^, some genes including *TP53*, *KEAP1*, *RAB34* and *WNT3A* were expressed more in the TCGA cancer groups with *TM4SF5* overexpression (of LIHC, KIRC, COAD or STAD) than in TCGA cancer groups without *TM4SF5* overexpression (of BRCA, SKCM, ACC or UCEC), whereas *SNX1*, *GAB1* and *GSK3B* were expressed in the opposite pattern (Fig. 2f). It is also worth highlighting that the cancer types without TM4SF5 expression changes did not show dramatic changes in the DEMRGs (Fig. 2f). Such nutrient type-dependent FITC-ALB uptake mediated by TM4SF5 forms appeared to be related to mTOR and AMPK activity, indicating that energy-limited stressful conditions driven by an SFM condition were recovered by GLU, GLU + ALB or GLU + FBS replenishment in TM4SF5_WT_ cells. However, no such pattern was observed in TM4SF5_A132V_ cells (Fig. 2g). ALB treatment of Huh7_KO3#2_-TM4SF5_WT_ cells in GLU + SFM led to notably increased FITC-ALB uptake, whereas control (TM4SF5-null) Huh7_KO3#2_ cells and TM4SF5_A132V_ cells showed no dose-dependent changes in uptake upon ALB treatment (Fig. 2h). Meanwhile, (GLU-free) SFM with GLU alone led to an increased response in TM4SF5_A132V_ cells but not in control or TM4SF5_WT_ (nonsignificance, *P* = 0.0879) cells (Fig. 2i). ALB alone or ALB + GLU treatment substantially increased FITC-ALB uptake in TM4SF5-overexpressing SNU449 cells or endogenously TM4SF5-expressing HepG2 cells, but not in TM4SF5-lacking SNU449 or TM4SF5-suppressed HepG2 cells (Fig. 2j and Supplementary Fig. 4). Furthermore, in SNU449 cells lacking endogenous TM4SF5, changes in the cellular filopodia-like processes at the edges depended on TM4SF5_WT_ expression and ALB replenishment of SFM (Supplementary Fig. 4a), as shown in Huh7 cell variants (Fig. 1). Thus, TM4SF5-mediated morphological changes in hepatocytes might be involved in extracellular albumin uptake.

### TM4SF5_WT_ promotes PIP_3_-mediated macropinocytosis depending on nutrient replenishment

**Fig. 3:**
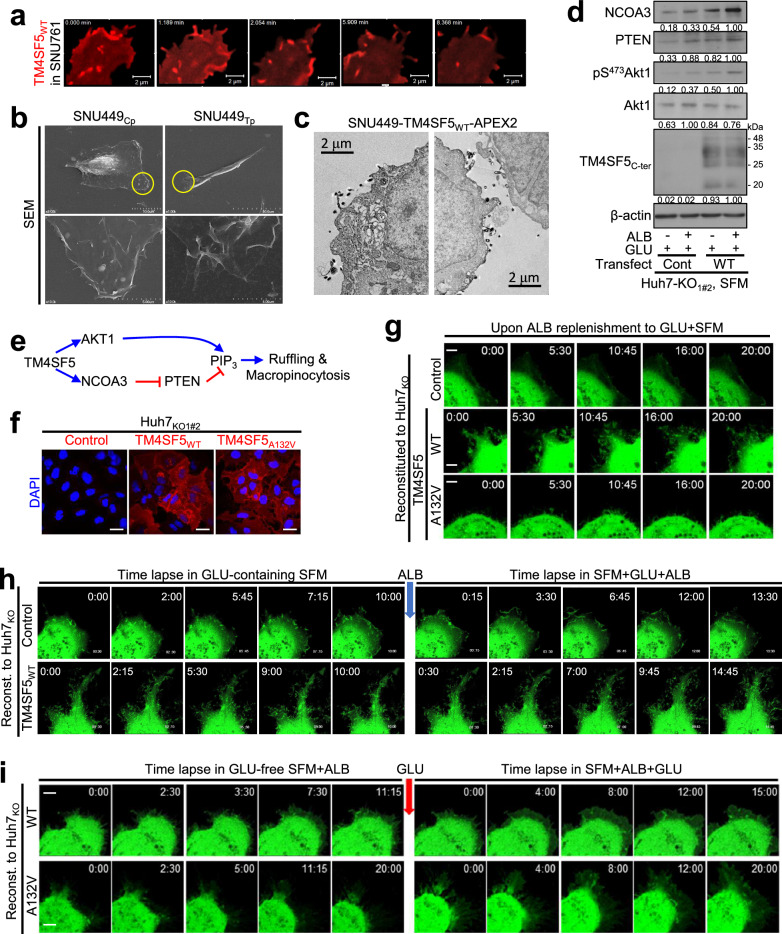
TM4SF5 WT promoted PIP_3_-mediated macropinocytosis depending on nutrient replenishment. **a** SNU761 hepatocytes stably transfected with pmCherry-TM4SF5_WT_ were live imaged in normal culture media at 37 °C and 5% CO_2_. **b** TM4SF5-negative SNU449_Cp_ or TM4SF5-positive SNU449_Tp_ cells in normal culture conditions were processed for scanning electron microscopy. The yellow circles were enlarged for membrane ruffling (bottom). **c** SNU449 cells exogenously transfected with TM4SF5_WT_ conjugated with APEX2 were processed for transmission electron microscopy^33^. Note that membrane edges showed ruffling with TM4SF5-APEX2-positive dark dots. **d** Huh7_KO_ cell variants replenished with GLU alone or ALB + GLU to SFM for 16 h were processed for immunoblotting. The band intensities measured by ImageJ software were normalized to those of the loading control. **e** A schematic of possible molecular linkage from TM4SF5 to PIP_3_ for ruffling and macropinocytosis. **f**, **g** Huh7-KO_1#2_ cells transduced with control, TM4SF5_WT_ or TM4SF5_A132V_ lentivirus were replated on collagen I and cells were then imaged to observe nondifferential TM4SF5 expression (**f**) or transiently transfected with pEGFP-AKT-PH for 24 h and manipulated as above for serum starvation and nutrient replenishment, before live imaging (15 s intervals for 20 min) around cellular edges (**g**). Representative images were collected and are presented. **h**, **i** Huh7_KO_ cell variants were manipulated as in **g** with the indicated nutrients: after live imaging for specific durations, chambers on the microscope stage were replenished with ALB (3.6 mg/ml, **h**) or GLU (25 mM, **i**), and live imaging continued. Data represent three independent experiments. See also Supplementary Movies 1–4.

As we observed nutrient-dependent actin remodeling (Fig. 1) and an apparent correlation between FITC-ALB uptake and DEMRGs (Fig. 2) in TM4SF5-positive hepatocytes, we examined whether macropinocytosis-related morphological changes depend on extracellular nutrient supply. First, we often observed that TM4SF5 at membrane edges dynamically changed shape, similar to ruffles with filopodia-like processes (Fig. 3a and Supplementary Movie 1). We then observed, via electron microscopy, that membrane edges of TM4SF5_WT_-expressing SNU449 (SNU449_Tp_) cells showed more aggressive ruffling and architecture for the engulfing process than did TM4SF5-null SNU449_Cp_ hepatocytes (Fig. 3b,c). In addition, immunoblots showed increased Akt1 activity that was dependent on TM4SF5 expression and additional ALB replenishment of serum-starved hepatocytes, which might reflect PIP_3_ accumulation (Fig. 3d,e). As macropinocytosis is promoted by PTEN deficiency^15^ that also reduces PIP_3_ levels, we investigated whether PIP_3_-mediated macropinocytosis of hepatocytes in SFM was differentially regulated by TM4SF5 forms and whether such TM4SF5-dependent effects were affected by ALB and/or GLU replenishments, using fluorescence imaging of cell surfaces presumably enriched with PIP_3_ following transfection of pEGFP-AKT-PH (the GFP-conjugated PH domain of AKT is capable of binding PIP_3_ accumulated at plasma membranes^47^). First, Huh7_KO1#2_ cells stably expressing control or TM4SF5 WT or A132V mutant forms were confirmed via fluorescence imaging (Fig. 3f). Live imaging of cells transfected with the pEGFP-AKT-PH construct immediately after ALB replenishment in GLU + SFM showed morphological changes or ruffles around the cell surface, which were markedly less observable in control cells (Supplementary Movie 2). TM4SF5_WT_ cells showed dynamic ruffles (Supplementary Movie 3), but A132V mutant cells showed slightly less ruffling (Fig. 3g and Supplementary Movie 4). When membrane ruffles of control or TM4SF5_WT_ cells were live imaged before and after ALB replenishment in GLU + SFM, WT cells showed ruffles before and increased ruffling after ALB replenishment, whereas control cells responded less dynamically (Fig. 3h). When membrane ruffles of TM4SF5_WT_ or TM4SF5_A132V_ mutant cells were live imaged before and after GLU replenishment of ALB-containing (but GLU-free) SFM, WT cells showed an active baseline and dynamically responding ruffles after GLU treatment, whereas TM4SF5_A132V_ cells showed more aggressive ruffling after GLU treatment (Fig. 3i). Therefore, different TM4SF5 forms (WT or A132V mutant) appear to differentially require nutrients (ALB alone or GLU + ALB, respectively) for macropinocytosis in SFM. Thus, TM4SF5-mediated changes in boundary morphology and ruffling appear to involve PIP_3_-dependent ALB uptake depending on TM4SF5 and extracellular nutrients.

### Mitochondrial ATP-linked respiration is followed by TM4SF5-mediated ALB uptake

**Fig. 4:**
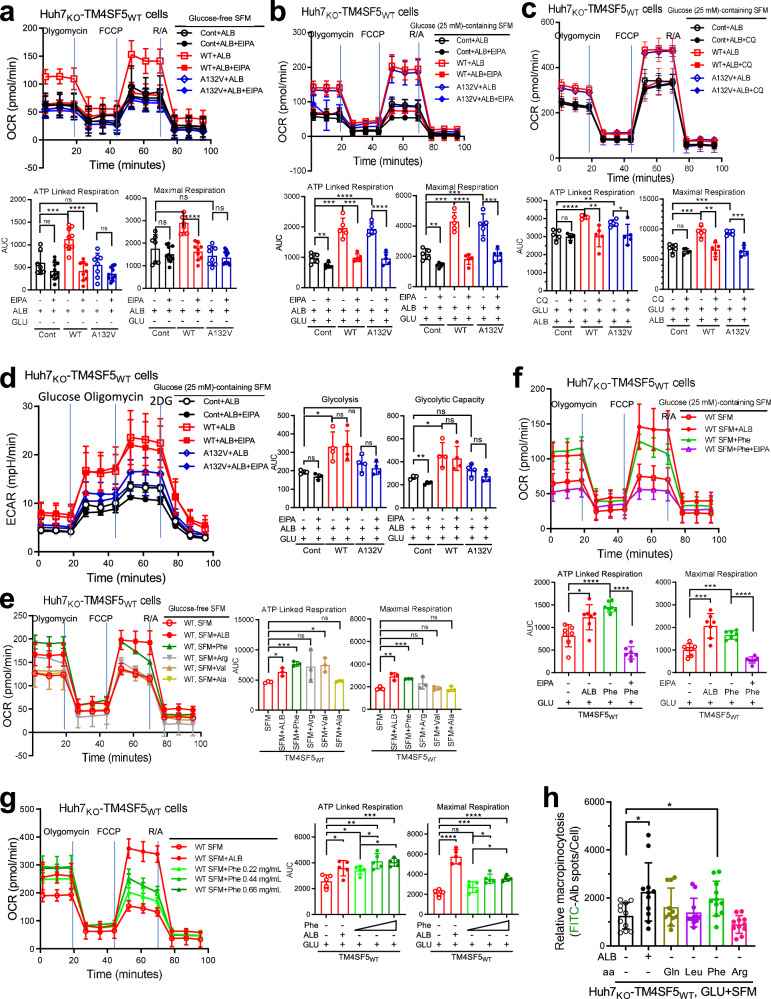
Mitochondrial ATP-linked respiration was followed by TM4SF5-mediated ALB uptake. Huh7-KO_3#309_ cells were transduced using a lentivirus for control empty vector, TM4SF5_WT_ or TM4SF5_A132V_ expression. On the basis of OCR or ECAR analyses, ATP-linked and maximal respiration as parameters of mitochondrial function and glycolysis or glycolytic capacity for glycolytic functions were calculated and plotted at mean ± s.e.m. values. Each data point in the graphs from the OCR and ECAR measurements represent a mean value of a triplicate experiment. **a**–**d** Cells were then replenished with ALB to GLU-free SFM (**a**) or to GLU-containing SFM (**b**) without or with EIPA (10 μM, **a**, **b** and **d**) or CQ (25 μM, **c**) treatment for 15 h before a mitochondria stress test to measure their OCR (**a**–**c**) or before measurement of ECAR (**d**). **e**, **f** Cells were replenished with single amino acids to GLU-free (**e**) or GLU-containing (**f**) SFM with or without EIPA treatment for 15 h, before OCR analysis. **g** Cells were replenished with Phe at different concentrations to GLU + SFM for 15 h before OCR analysis. **h** Huh7_KO_-TM4SF5_WT_ cells were replenished with or without ALB alone or each amino acid to GLU + SFM for 15 h. Relative FITC-ALB uptake was measured in replated on collagen I as an index of macropinocytosis. *P* values were calculated via one-way ANOVA or unpaired Student’s *t*-tests, and *P* < 0.05 was considered statistically significant. *, **, *** and **** depict *P* < 0.05, 0.01, 0.001 and 0.0001, respectively. Data represent three independent experiments. See also Supplementary Fig. 5.

We next explored whether macropinocytosis-mediated ALB uptake is linked to cellular bioenergetics. The OCR, as an index of mitochondrial respiration for ATP synthesis, was measured in cells receiving ALB replenishment in GLU-free SFM with or without treatment with EIPA, a macropinocytosis inhibitor. Unlike control Huh7_KO_ or Huh7_KO_-TM4SF5_A132V_ mutant cells, Huh7_KO_-TM4SF5_WT_ cells showed efficient increases in ATP-linked and maximal respiration with ALB replenishment, which were abolished by EIPA treatment (Fig. 4a). Meanwhile, H^+^-ATPase inhibition using bafilomycin A did not alter the ALB-mediated OCR of control or TM4SF5_WT_ cells, but blocked the ALB-mediated increase in the OCR of TM4SF5_A132V_ cells (not significant, *P* = 0.0841 for maximal respiration) (Supplementary Fig. 5a). However, upon ALB replenishment of GLU + SFM, Huh7_KO_-TM4SF5_A132V_ mutant cells also showed efficient increases in respiration that were abolished by EIPA treatment, similar to WT cells (Fig. 4b). Next, we examined whether inhibition of lysosomal protein degradation via chloroquine (CQ) treatment could also block the effects of TM4SF5-mediated ALB uptake. CQ treatment abolished the increased respiration in WT or A132 mutant cells (Fig. 4c). When the ECAR was measured as a glycolytic index, Huh7_KO_-TM4SF5_WT_ but not TM4SF5_A132V_ mutant cells showed notably increased glycolysis and glycolytic capacity compared with Huh7_KO_ control cells, independent of EIPA treatment (Fig. 4d). These observations indicate that ALB uptake by TM4SF5-mediated macropinocytosis might be catabolized for mitochondrial ATP synthesis.

We next examined whether single amino acids mimicked the effects of ALB replenishment on mitochondrial respiration rates when each amino acid was treated at the concentration corresponding to its composition in ALB. When different amino acids were separately administered to Huh7_KO_-TM4SF5_WT_ cells in GLU-free SFM, Phe produced efficient ATP-linked respiration similar to ALB (Fig. 4e). However, administration of other amino acids less efficiently increased ATP-linked respiration and/or maximal respiration to levels not sufficiently high to mimic ALB; Ser, His and Ile appeared to slightly mimic ALB-mediated effects (Fig. 4e and Supplementary Fig. 5b–d). Furthermore, ATP-linked respiration of TM4SF5_WT_ cells was also substantially increased upon Phe treatment of GLU + SFM, similar to ALB, and was abolished by EIPA treatment, indicating that extracellular Phe could be taken up via macropinocytosis (Fig. 4f). In addition, Phe-mediated ATP-linked respiration of TM4SF5_WT_ cells in GLU + SFM increased in a dose-dependent manner (Fig. 4g). Meanwhile, upon single amino acid treatment of TM4SF5_WT_ cells in SFM, macropinocytosis was notably increased by Phe, similar to ALB, but not by other amino acids such as Gln, Leu and Arg (Fig. 4h). These observations may indicate that ALB degradation resulting in free Phe in lysosomes could be used for ATP-linked respiration. Therefore, TM4SF5-mediated ALB uptake might have potential for ATP synthesis.

### TM4SF5-mediated ALB uptake is linked to enhanced migratory potential

**Fig. 5:**
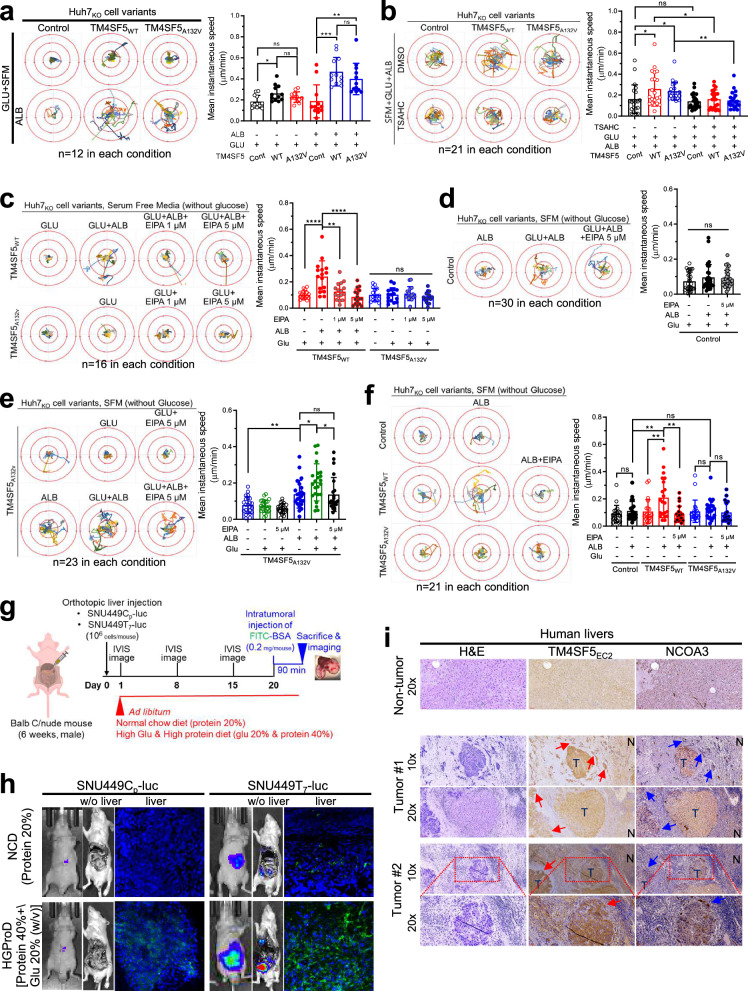
TM4SF5-mediated ALB uptake was linked to enhanced metastatic potential. **a** Control Huh7_KO_, Huh7_KO_-TM4SF5_WT_ or Huh7_KO_-TM4SF5_A132V_ cell variants replated on collagen I with or without replenishment of ALB (3.6 mg/ml) to GLU (25 mM) + SFM for 15 h were analyzed for cell migration via tracking of individual cell movement for 17 h using a time-lapse microscope (12 cells per condition, left). The mean instantaneous speed per condition was calculated using MetaMorph software and presented (in μm/min, right). The dots indicate a mean value of the instantaneous speed calculated from the total migration distance of a cell for a measurement interval of 20 min. During the measurement period of 17 h, many instantaneous speed values (3 times per h × 17 h) were calculated for the mean value of a cell. **b**–**f** Huh7_KO_ cell variants manipulated as in **a** were treated with DMSO or TSAHC (5 μM) before cellular tracking for 16 h (**b**), with or without EIPA treatment at different concentrations before cellular tracking for 17 h (**c**–**f**) and calculation of the mean instantaneous speed. Each trajectory line depicts a trajectory of one individual cell. *P* values were calculated via one-way ANOVA or unpaired Student’s *t*-tests, and *P* < 0.05 was considered statistically significant. **g**, **h** A schematic for the analysis of in vivo macropinocytosis (**g**): six-week-old BALB/c-nude male mice were liver-orthotopically injected with TM4SF5-null SNU449_Cp_ or TM4SF5-overexpressing SNU449_T7_ cells stably transfected with luciferase (luc) constructs (10^6^ cells/injection/mouse); luciferase signal was measured from mice fed a NCD containing 20% protein or a HGProD containing 20% GLU and 40% protein (*n* > 8) until day 20; on day 20, FITC-BSA (0.2 mg/mouse) was intratumorally injected, and 90 min later mice were killed for imaging of green signal in the liver; and representative images of luciferase in animals and FITC-BSA signals in liver tissue were obtained using IVIS Spectrum (**h**). **i** Liver tissues from patients with HCC (*n* = 7) were processed for H&E staining or immunohistochemistry. Data represent three independent experiments. See also Supplementary Fig. 6.

We next explored the biological importance of TM4SF5-mediated ALB uptake for ATP synthesis. As cell migration involves dynamic cytoskeletal rearrangements that require mitochondrial energetics^48^, we analyzed the migratory capacity by tracking single cells via imaging under various experimental conditions. In GLU + SFM, Huh7_KO_-TM4SF5_WT_ cells showed increased migration unlike Huh7_KO_-control or Huh7_KO_-TM4SF5_A132V_ mutant cells, and upon additional ALB replenishment, TM4SF5_WT_ and TM4SF5_A132V_ mutant cells in GLU + SFM showed notably increased migration compared with that of control cells (Fig. 5a). Such TM4SF5-mediated migration upon ALB replenishment of GLU + SFM was abolished by TSAHC treatment (a specific TM4SF5 inhibitor^29^; Fig. 5b). Furthermore, TM4SF5_WT_ cell migration enhanced by ALB replenishment of GLU + SFM was also abolished by EIPA treatment, whereas migration of TM4SF5_A132V_ mutant cells upon replenishment of (GLU-free) SFM with GLU alone did not show further changes after GLU + EIPA treatment (Fig. 5c). Upon GLU + ALB treatment, control cells did not respond to EIPA treatment (Fig. 5d). Migration of TM4SF5_A132V_ mutant cells was not changed by GLU alone but was increased by ALB alone or GLU + ALB replenishment, and this effect was abolished by EIPA treatment (Fig. 5e). In SFM without basal GLU, TM4SF5_WT_ cells, but not control or TM4SF5_A132V_ mutant cells, exhibited migration that was sensitive to ALB and abolished by EIPA treatment (Fig. 5f).

In addition to the in vitro cell migration assay, we determined whether the TM4SF5-mediated effects were also valid in in vivo animal models. Male nude mice with liver orthotopic injection of HCC cells containing a luciferase reporter without (SNU449C_p_-luc) or with (SNU449T_7_-luc) TM4SF5 expression were given ad libitum access to a NCD containing 20% protein or an HGProD containing 20% GLU (w/v) and 40% protein for 20 days. On day 20, FITC-BSA was intratumorally injected to obtain a clear macropinocytotic fluorescence signal and 90 min later, liver tissue was collected and imaged using an IVIS animal imaging system (Fig. 5g and Supplementary Fig. 6a). Compared with SNU449C_p_-luc injection, SNU449T_7_-luc-injected mice fed an NCD showed increased tumor size or invasive multifocality in the liver (Fig. 5h, top, and Supplementary Fig. 6a), and SNU449T_7_-luc-injected mice fed an HGProD showed a larger tumor size (Fig. 5h, bottom, and Supplementary Fig. 6a). When FITC-BSA macropinocytosis was imaged, SNU449T_7_ cell-injected mice showed increased macropinocytosis (Fig. 5h, right) and even higher macropinocytosis with the HGProD than with the NCD (Fig. 5h, bottom, and Supplementary Fig. 6b,c). These results indicate that TM4SF5-positive liver cancer cells could uptake more FITC-BSA in mice fed an HGProD. Furthermore, TM4SF5-mediated luciferase signal might be positively correlated with enhanced macropinocytosis, indicating that TM4SF5 plays a role in intrahepatic metastasis, resulting in expanded tumor size or multifocality. Regarding this correlation, human patients with HCC showed higher TM4SF5 expression in tumor regions and metastatically invasive tumor cells (arrows) than in nontumor regions (Fig. 5i). Therefore, hepatocyte TM4SF5 might lead to intrahepatic micro-metastasis, expanded tumor size or multifocality via enhanced macropinocytosis resulting from ALB uptake and energetic increases.

### TM4SF5-mediated macropinocytosis appears to involve cytosolic NCOA3 stabilization

**Fig. 6:**
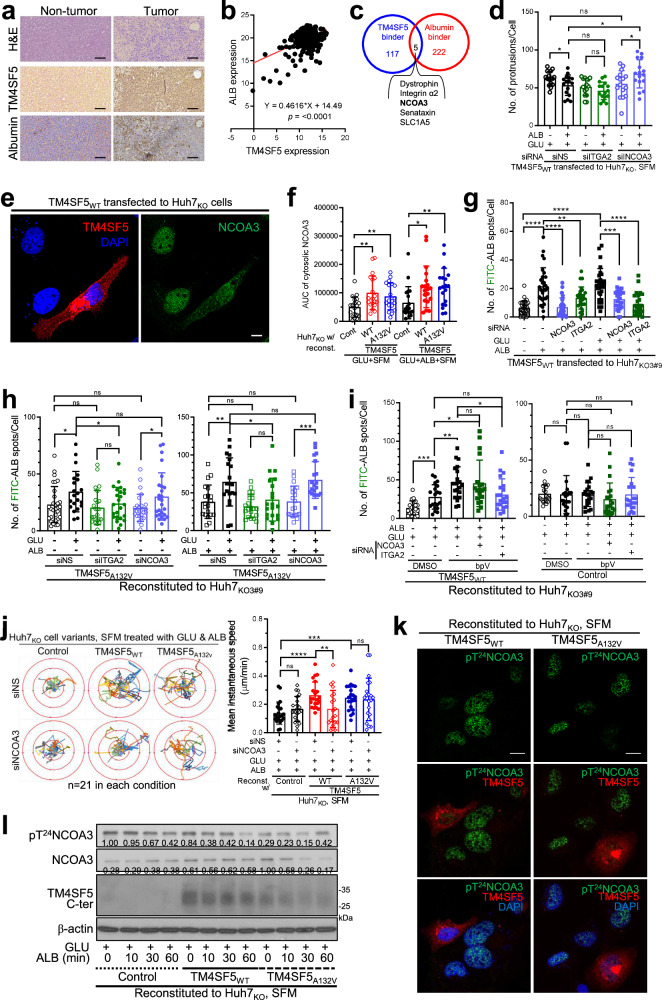
TM4SF5-mediated macropinocytosis involves cytosolic NCOA3 stabilization. **a** Liver tissues from patients with HCC (*n* = 7) were processed for H&E staining or immunohistochemistry. **b** Analysis of co-expression between TM4SF5 and ALB in the LIHC (HCC) set from the TCGA database. **c** A Venn diagram showing protein binding to TM4SF5 (ref. ^21^) or ALB (https://thebiogrid.org/106715/summary/homo-sapiens/alb.html)^49^. **d** Huh7-KO_1#2_ cells stably transfected with TM4SF5_WT_-HA cDNA were transfected with siRNA against control (NS), ITGA2 or NCOA3 sequences for 24 h and then replenished with GLU (25 mM) alone or ALB + GLU (3.6 mg/ml and 25 mM, respectively) to SFM for 15 h before analysis of the number of protrusions per cell. Each data point depicts a value from one individual cell. **e** Huh7_KO_-TM4SF5_WT_-HA cells were replenished with ALB + GLU to SFM for 15 h and processed for immunofluorescence. **f** Huh7_KO_ cell variants were replenished with GLU alone or GLU + ALB to SFM for 15 h, before immunofluorescence for NCOA3 with DAPI co-staining. **g**–**l** Huh7_KO_ cell variants were transfected with indicated siRNA for control sequence (siNS) or siRNA against specific sequences (Table 1) for 24 h and then replenished with nutrients as indicated for 15 h before analysis of FITC-ALB spots (green) per (red fluorescent) (**g**–**i**), cellular tracking for 17 h (**j**), immunofluorescence (**k**) or immunoblotting (**l**). Alternatively, cells were treated with DMSO or the PTEN inhibitor bpV (Phen) during nutrient replenishment for 15 h before analysis on FITC-ALB spots per cell. *P* values were calculated via one-way ANOVA or unpaired Student’s *t*-tests, and *P* < 0.05 was considered statistically significant. Data represent three independent experiments. See also Supplementary Figs. 7 and 8.

We next examined which molecules were involved in TM4SF5 relevance to ALB level in patients with HCC. In addition to TM4SF5-positive liver tissue from patients with HCC showing obvious ALB levels (Fig. 6a), patients with liver cancer (LIHC) from TCGA datasets showed a positive correlation between TM4SF5 and hepatic ALB levels (Fig. 6b). We next explored which molecules or pathways could be involved in TM4SF5-mediated effects. Interestingly, we found five molecules commonly considered to bind to TM4SF5 or ALB when data from proteomic analysis of TM4SF5 (ref. ^21^) and a previous study on BSA-binding proteins were compared^49^, including nuclear receptor coactivator 3 (NCOA3, also known as SRC3/RAC3) with intrinsic histone acetyltransferase activity^50^ (Fig. 6c). Among the five genes, analyses of the TCGA + GTXe^44^ datasets and GSE14520 patients with HCC showed that NCOA3 could be functionally related to TM4SF5 and positively higher in tumor tissues, unlike ALB levels (Supplementary Fig. 7a). We further found that NCOA3 is important for TM4SF5-mediated effects on protrusive processes formation; ALB-treated TM4SF5_WT_ cells in GLU + SFM showed fewer protrusions than nontreated cells, which was ameliorated by suppression of NCOA3 but not integrin α2 (ITGA2) (Fig. 6d). Meanwhile, suppression of other molecules such as dystrophin or SLC1A5 did not alter basal or ALB-promoted FITC-ALB uptake (Supplementary Fig. 7b).

We next investigated how NCOA3 participates in TM4SF5-mediated effects. During immunofluorescence imaging, TM4SF5_WT_ transfectants showed increased NCOA3 translocation and stabilization in the cytosol, unlike untransfected Huh7_KO_ cells (Fig. 6e). When the area under the curve of NCOA3 fluorescence intensity in the cytosol outside the nucleus was examined (Supplementary Fig. 7c), both TM4SF5_WT_ and TM4SF5_A132V_ expression resulted in increased cytosolic NCOA3 staining in GLU + SFM with or without ALB replenishment (Fig. 6f). ALB alone or ALB + GLU replenishment in TM4SF5_WT_ cells in SFM led to increased FITC-ALB uptake, whereas these effects were abrogated by additional suppression of NCOA3 or ITGA2 (Fig. 6g). Even further suppression of ITGA4 or ITGA5 did not block the increase in FITC-ALB uptake upon ALB + GLU replenishment in TM4SF5_WT_ cells in SFM (Supplementary Fig. 7d). Holotomography imaging at the cellular edges of TM4SF5_WT_ cells showed active ruffles promoted by ALB + GLU replenishment of SFM compared with those in Huh7_KO_ control cells, which was abolished by NCOA3 suppression (Supplementary Fig. 8, red arrows). Meanwhile, GLU alone or ALB + GLU replenishment of TM4SF5_A132V_ mutant cells in SFM increased FITC-ALB uptake, which was abolished by suppression of ITGA2 but not NCOA3. However, TM4SF5_A132V_ mutant cells in SFM without or with ALB-only replenishment did not show changes in uptake independent of suppression (Fig. 6h). We next examined whether NCOA3 with histone acetyltransferase activity might affect PIP_3_-mediated macropinocytosis via regulation of PTEN acetylation and inactivation. Interestingly, FITC-ALB uptake in TM4SF5_WT_ cells in GLU + SFM increased upon ALB replenishment, which was further increased by the specific PTEN inhibitor bpV (Phen)^51^, indicating that TM4SF5-mediated ALB uptake might be modulated by PTEN activity (Fig. 6i, left). Furthermore, the PTEN inhibition-mediated FITC-ALB uptake of TM4SF5_WT_ cells was abolished by suppression of ITGA2 but not NCOA3 (Fig. 6i, left), suggesting roles of ITGA2 in FITC-ALB uptake independent of NCOA3–PTEN linkage. Meanwhile, TM4SF5-negative (that is, Huh7_KO_) control cells showed nonsignificant changes in FITC-ALB uptake upon nutrient replenishment, bpV (Phen) treatment or ITGA2 or NCOA3 suppression (Fig. 6i, right). Interestingly, cellular migration and instantaneous speed were increased with ALB + GLU replenishment of SFM in TM4SF5_WT_ cells compared with those in control cells. However, these effects were abolished by NCOA3 suppression, and no effects on TM4SF5_A132V_ mutant cells were observed upon NCOA3 suppression (Fig. 6j). Phosphorylation of the Thr 24 residue in NCOA3 (that is, pT^24^NCOA3, which is required for nuclear translocation^52^) appeared reduced and was insufficient for nuclear translocation in TM4SF5_WT_ and TM4SF5_A132V_ mutant cells (Fig. 6k). TM4SF5_WT_ cells showed gradually reduced pT^24^NCOA3 levels and sustained NOCA3 levels over time after ALB replenishment of GLU + SFM, unlike TM4SF5_A132V_ cells (Fig. 6l). Thus, the importance of cytosolic NCOA3 expression for TM4SF5-mediated effects appeared more obvious in TM4SF5_WT_ cells.

### TM4SF5-mediated protein complex formation with NCOA3 and PTEN leads to NCOA3 stabilization and PTEN inactivation

**Fig. 7:**
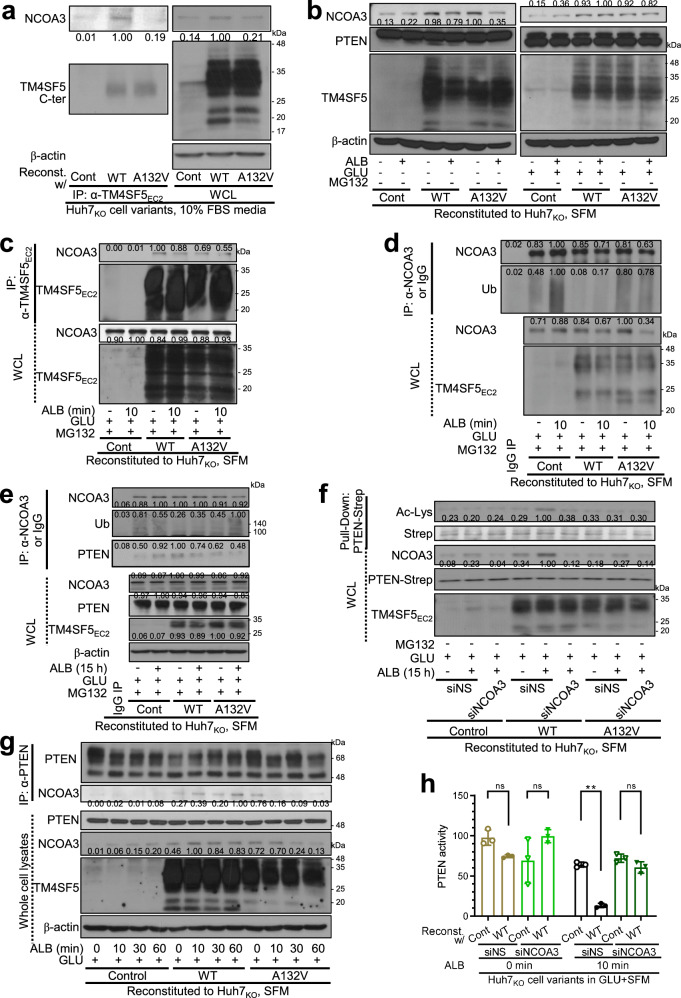
TM4SF5-mediated complex formation with NCOA3 and PTEN led to NCOA3 stabilization and PTEN inactivation. **a** Huh7_KO_ cell variants in normal culture condition with 10% FBS were were collected for whole-cell lysates (WCL), before co-immunoprecipitation (IP) using anti-TM4SF5_EC2_ antibody. **b** Huh7_KO_ cell variants were replenished with nutrients as indicated for 15 h and collected before immunoblots for the indicated molecules**. c**–**g** Huh7_KO_ cell variants were or were not replenished with ALB to GLU + SFM for 0, 10, 30 or 60 min with or without MG132 (10 μM) treatment to block NCOA3 degradation: WCL were processed by IP using normal IgG (IgG), anti-TM4SF5_EC2_ polyclonal antibody (**c** and **d**, before immunoblotting for NCOA3 and either TM4SF5_EC2_ (**c**) or ubiquitin (**d**)), anti-NCOA3 rabbit monoclonal antibody (**e**), strep (**f**) or anti-PTEN rabbit monoclonal antibody (**g**). Immunoprecipitates were then immunoblotted in parallel to WCL and in some cases, cells were transfected with siNS or siNCOA3 for 24 h before nutrient replenishment (**f**). Ratio values of band intensities of certain immunoblots measured by using ImageJ software were normalized to those of the loading control or the total form of the molecule are presented. **h** Huh7_KO_ cell variants were transfected with siNS or siNCOA3 for 24 h. ALB replenishment to GLU + SFM was performed for 0 or 10 min before the PTEN activity assay. *P* values were calculated via one-way ANOVA or unpaired Student’s *t*-tests, and *P* < 0.05 was considered statistically significant. Data represent three independent experiments.

We next explored how NCOA3 may be involved in PIP_3_-mediated macropinocytosis in TM4SF5_WT_ hepatocytes. First, we examined whether TM4SF5_WT_ bound to NCOA3 because NCOA3 might be stabilized via association with TM4SF5 upon TM4SF5-mediated cytosolic translocation. Unlike TM4SF5_A132V_ mutant cells, TM4SF5_WT_ cells in normal 10% FBS-containing culture bound to NCOA3 (Fig. 7a). Compared with NCOA3 levels in control cells, TM4SF5_WT_ cells showed sustained levels upon replenishment of SFM with ALB alone or ALB + GLU, whereas TM4SF5_A132V_ mutant cells showed slightly reduced levels (Fig. 7b). These may indicate a differential nutrient prerequisite for NCOA3 stabilization (as shown in Fig. 3d). Furthermore, when cells were treated with MG132 to enrich NCOA3 without proteasomal degradation, both TM4SF5_WT_ and TM4SF5_A132V_ mutant cells showed TM4SF5 bound to NCOA3 without dramatic changes upon replenishment of SFM with GLU alone or ALB + GLU (Fig. 7c). This observation suggests that the association of TM4SF5 with cytosolically translocated NCOA3 via nutrient replenishment might be linked to its stabilization. Furthermore, TM4SF5_A132V_ mutant cells showed increased NCOA3 ubiquitination upon ALB + GLU replenishment of SFM, unlike TM4SF5_WT_ cells (Fig. 7d). Furthermore, TM4SF5_WT_ cells showed greater NCOA3 binding to PTEN than did control or TM4SF5_A132V_ cells in replenishment of SFM with GLU alone; upon ALB + GLU replenishment, NCOA3 binding to PTEN in control cells increased but reduced in TM4SF5_WT_ or TM4SF5_A132V_ cells (Fig. 7e). Interestingly, TM4SF5_WT_ cells with less ubiquitinated NCOA3 showed increased NCOA3 binding to PTEN in the GLU + SFM condition but decreased binding in the ALB + GLU + SFM condition, whereas TM4SF5_A132V_ mutant cells with more ubiquitinated NCOA3 bound to PTEN, which was sustained even in the ALB + GLU + SFM condition (to a level lower than that of TM4SF5_WT_ cells) (Fig. 7e). As NCOA3 has histone acetyltransferase activity^50^, we examined whether NCOA3-bound PTEN might be acetylated at lysine residues. As expected, PTEN in TM4SF5_WT_ cells showed more lysine acetylation with sustained NCOA3 expression in the ALB + GLU + SFM condition than in the GLU + SFM condition, whereas TM4SF5_A132V_ mutant cells showed reduced PTEN acetylation with a reduced NCOA3 level upon additional ALB replenishment to GLU + SFM (Fig. 7f, lanes 4, 5, 7 and 8). Again, NCOA3 suppression reduced acetylated PTEN in TM4SF5_WT_ cells but not in TM4SF5_A132V_ mutant cells (Fig. 7f, lanes 6 and 9). Over time, after ALB replenishment, TM4SF5_WT_ cells showed sustained binding of NCOA3 to PTEN, unlike KO control or A132V mutant cells (Fig. 7g). PTEN is known to be inactivated upon acetylation at lysine residues by histone acetyltransferases, such as p300/CBP-associated factor (PCAF)^53^. When we measured PTEN activity upon ALB replenishment of GLU + SFM, TM4SF5_WT_ cells showed decreased PTEN activity depending on NCOA3 expression (Fig. 7h). Therefore, TM4SF5-dependent cytosolic location of NCOA3 might be possible via an association between TM4SF5 and NCOA3, leading to NCOA3 stabilization and further acetylation and inactivation of PTEN (also bound to NCOA3), eventually leading to PIP_3_ accumulation upon ALB treatment of TM4SF5_WT_ cells in GLU + SFM.

**Fig. 8:**
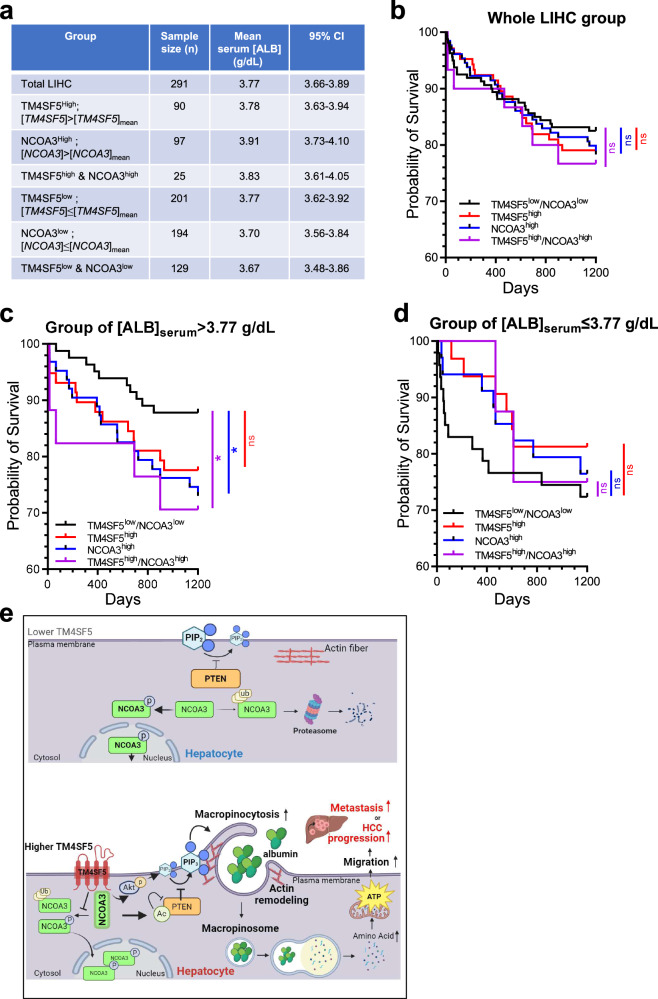
Clinical importance of *TM4SF5* and *NCOA3* mRNA expression and serum ALB levels of TCGA patients with LIHC in terms of survival probability. **a** The LIHC patient group from the public TCGA dataset (*n* = 291 with serum ALB level, [ALB]_serum_) showed a mean [ALB]_serum_ of 3.77 g/dl (that is, 37.7 mg/ml). Subgroups depending on the expression levels of *TM4SF5* and *NCOA3* mRNA showed different [ALB]_serum_ levels. **b** The entire LIHC patient group (*n* = 364 including patients without [ALB]_serum_ information) was analyzed for the probability of survival depending on the expression levels of *TM4SF5* and *NCOA3* mRNA. **c**, **d** The LIHC group was subclassified into [ALB]_serum_ >3.77 g/dl (**c**) or [ALB]_serum_ ≤3.77 g/dl (**d**). The subgroups were further subclassified into TM4SF5^low^/NCOA3^low^ (control), TM4SF5^high^, NCOA3^high^ or TM4SF5^high^/NCOA3^high^ categories to analyze the probability of survival depending on their mRNA expression levels. The survival probability of patient groups was analyzed to 1,200 days for statistical comparisons. *P* values were calculated by one-way ANOVA. **e** Schematic of the working model: in normal hepatocytes without notable TM4SF5 expression, extracellular ALB might not be efficiently uptaken for cellular energetics due to less macropinocytosis, supported by higher PTEN lipid phosphatase activity via greater NCOA3 localization into the nucleus or degradation in cytosol. Meanwhile, in cancerous hepatocytes with enhanced TM4SF5 expression, TM4SF5 binds to and promotes cytosolic stabilization of NCOA3, leading to an association with and acetylation of PTEN, which could eventually become inactive for PIP_3_ accumulation on membranes to allow efficient macropinocytosis-mediated uptake of extracellular ALB. ALB may then be degraded for mitochondrial ATP synthesis, leading to greater energetics for cell migration, presumably during intrahepatic metastasis for tumor nodule expansion or multifocality. See also Supplementary Fig. 7.

Although patients with HCC generally show lower serum ALB levels that are associated with poor survivals^3^, TM4SF5-mediated patients with HCC (that is, patients with HCC with TM4SF5 increases over nontumor samples) showed increased ALB mRNA and protein expression in the liver (Fig. 6a,b). Patients with HCC in TCGA dataset (*n* = 291 out of total LIHC 369, due to the availability of serum ALB levels, [ALB]_serum_) showed a mean [ALB]_serum_ of 3.77 g/dl (at the pre-resection stage; Fig. 8a). Finally, we grouped patients depending on their TM4SF5 and/or NCOA3 expression levels for survival probability analysis (Fig. 8b). Interestingly, TM4SF5 expression alone was not importantly related to survival, either in the entire LIHC patient cohort (*P* = 0.5752), or in the NCOA3^high^ or TM4SF5^high^/COA3^High^ groups (Fig. 8b). Meanwhile, among patients with [ALB]_serum_ >3.77 g/dl, the NCOA3^high^ or TM4SF5^high^/NCOA3^high^ groups showed significantly poorer clinical outcomes compared with those of the TM4SF5^low^/COA3^low^ group, and the TM4SF5^high^ group showed a trend toward a poor clinical outcome (nonsignificant, *P* = 0.0925; Fig. 8c). However, *TM4SF5* mRNA expression was nortably increased in tumor samples from patients with HCC compared with in nontumor samples, unlike *ALB* mRNA levels (Supplementary Fig. 7a). Among patients with [ALB]_serum_ ≤3.77 g/dl, no groups showed clinical outcomes that were substantially different from those of the TM4SF5^low^/COA3^low^ group (Fig. 8c, d). Therefore, a positive link between TM4SF5 and NCOA3 might lead to poor survival, presumably involving high [ALB]_serum_ levels. Therefore, it is likely that TM4SF5_WT_-mediated NCOA3 stabilization in the cytosol may lead to tumor nodule size and number expansion and HCC progression via TM4SF5-promoted ALB uptake, especially in an environment in which high [ALB]_serum_ may be available, presumably recapitulating earlier HCC development and expansion with less hepatocyte malignancy and functional loss.

## Discussion

This study reveals the roles of ALB uptake through macropinocytosis supported by PIP_3_ (Fig. 6) via TM4SF5–NCOA3–PTEN linkage (Fig. 7). TM4SF5-mediated cell surface morphological changes (Fig. 1) modulated ALB uptake into the cytosol (Fig. 2) via PIP_3_-mediated ruffling and/or macropinocytosis (Fig. 3). Then, ALB may be degraded to increase amino acid pools, eventually leading to increased mitochondrial ATP production (Fig. 4) to enhance cellular migration capacity (Fig. 5), presumably while intrahepatic micro-metastasis increases tumor nodule number or size and/or further metastasis (Fig. 8). TM4SF5_WT_ cells in SFM showed an increase in filopodia-like processes, which were inhibited by replenishment of ALB, a major component (40–60%) of blood^54^. Such morphological changes in membrane edges were inversely related to macropinocytosis and FITC-ALB uptake; increased process formation resulted in decreased macropinocytosis and FITC-ALB uptake. Hepatocyte TM4SF5 could bind to and cause cytosolic stabilization of NCOA3, a nuclear transcription coactivator with histone acetyltransferase activity that also associates with and acetylates PTEN, leading to PTEN inactivation^53^ and eventually resulting in enhanced phosphatidylinositol levels. Enrichment of PIP_3_ on the cell surface of TM4SF5_WT_ cells increased membrane ruffling, allowing macropinocytosis and ALB uptake. Such hepatocyte TM4SF5-mediated macropinocytosis upon supplying extracellular ALB was also observed in an in vivo animal model given an NCD or HGProD and liver-orthotopically xenografted TM4SF5-positive HCC cells. ALB taken up by TM4SF5_WT_ cells may have been catabolized for ATP generation via mitochondrial respiration, which could have enhanced cellular migration. Furthermore, liver tissue from patients with HCC showed increased TM4SF5 expression linked to increased cytosolic staining of NCOA3 in tumor lesions but not in nontumor regions. Thus, TM4SF5-promoted ALB uptake and catabolism were linked to ATP-linked respiration, supporting efficient cellular migration, which presumably allowed intrahepatic metastasis or achievement of multifocality during tumor nodule expansion or formation and cancer progression in the liver (Fig. 8e).

In addition to comparing TM4SF5-negative control and TM4SF5_WT_-positive hepatocytes, we compared TM4SF5_WT_ and TM4SF5_A132V_ mutant cells to understand the importance of TM4SF5 forms for protein metabolism. To eradicate the effects resulting from (even minimally) endogenous TM4SF5, we first knocked out the *TM4SF5* gene in Huh7 hepatocytes via a CRISPR–Cas9 approach, and the cells were then stably transduced or transfected with control, WT or A132V TM4SF5-expressing lentiviral particles or plasmids, resulting in multiple clones. In certain cases, we used different cell lines or variants for general aspects of TM4SF5-mediated effects. The TM4SF5_A132V_ mutant was compared with the WT form because the mutant exhibited a more elongated spindle-type morphology (data not shown) than the TM4SF5_WT_^16^. Additionally, this mutation occurred in Japanese patients with HCC (COSMIC sample ID: COSS2120737 and COSS2120738), although how HCC might develop via the A132V mutation remains unexplored. Thus, we speculated that the role of the A132V mutation in liver cancer development and progression might involve morphological changes. TM4SF5_WT_ cells showed differential mTOR and AMPK regulatory activity compared with TM4SF5_A132V_ mutant cells under different extracellular nutrient conditions. AMPK acts as a cellular energy sensor^55^, and mTOR is a central regulator that coordinates cellular nutrient levels^56^. TM4SF5_WT_ promotes mTOR activity via sensing physiological l-arginine levels in lysosomes^57^. Thus, TM4SF5-mediated morphological changes might be linked to metabolic activity in diverse nutrient-limited conditions. TM4SF5_WT_ cells showed TM4SF5-mediated effects including filopodia-like process formation, macropinocytosis, FITC-ALB uptake, ATP-linked respiration and efficient cellular migration upon ALB replenishment of (GLU-free) SFM. Meanwhile, TM4SF5_A132V_ mutant cells generally required both ALB and GLU for these effects, with GLU being a prerequisite for ALB-mediated effects. That is, there appears to be a different nutrient requirement for the TM4SF5-mediated morphological changes and ALB uptake between WT and A132V mutants. Protein or amino acid ingestion was previously found to support increases in blood insulin and glucagon, but not to increase blood GLU levels^58^, suggesting that protein ingestion may be irrelevant to blood GLU levels in certain metabolic scenarios. NCOA3 allowed the TM4SF5_WT_ cells to promote macropinocytosis-mediated ALB uptake, whereas both integrin α2 and NCOA3 appeared important for TM4SF5_A132V_-mediated effects, suggesting that the differential nutrient dependency is associated with different signaling activities or pathways. It will be interesting to examine how TM4SF5_WT_ and TM4SF5_A132V_ mutant cells require different nutrient types at the metabolic molecular levels to promote cancer progression in future studies.

TM4SF5 is involved in development of chronic liver diseases, including nonalcoholic steatohepatitis^59^, portal hypertension^19^ and HCC^17^. Hepatocytes produce ALB, and their malignancy leads to HCC. An inverse relationship is reported between [ALB]_serum_ levels and liver cancer risk^7^, and thus low serum ALB levels in patients with HCC are correlated with poor clinical outcomes^3^. Meanwhile, this study showed that TM4SF5-expressing hepatocytes can utilize extracellular ALB to produce ATP via mitochondrial respiration for cellular migration. *TM4SF5* mRNA levels in tumor samples of TCGA–LIHC dataset were positively correlated with hepatic *ALB* levels (Fig. 6b). However, the HCC patient dataset from TCGA showed that *ALB* mRNA levels in nontumor and paired tumor tissue samples were not notably different, with the tumor group showing a slightly lower level. However, a substantially lower *ALB* mRNA level was found in the cholangiocarcinoma tumor group than in the paired nontumor group (Ensembl ID: ENSG00000163631.16, GEPIA2) (Supplementary Fig. 7a). Thus, it is likely that TM4SF5-expressing hepatocytes in chronic liver diseases including HCC may still utilize extracellular ALB for gain of function during earlier pathological stages with less hepatocyte damage or malignancy. As explained by previous studies showing that [ALB]_serum_ levels are differentially linked to clinical outcomes depending on tumor nodule number and size^8–10^ (see ‘Introduction’ section), [ALB]_serum_ levels may affect multifocality in terms of tumor nodule number and size expansion and/or intrahepatic micro-metastasis. Thus, our observations may support that TM4SF5-expressing HCC cells can utilize extracellular ALB via macropinocytosis for ATP-linked respiration and cell migration, possibly leading to intrahepatic metastasis involving tumor nodule expansion or multifocality.

Spatio-temporal modification and trafficking of essential regulators between different cell compartments can be critical during HCC development and progression. As a tetraspan(in), TM4SF5 can form massive protein–protein complexes that traffic between different membranes^25,27^. While TM4SF5 promoted extracellular ALB uptake, it also promoted NCOA3 stabilization in the cytosol, which was associated with PIP_3_-mediated membrane ruffling and macropinocytosis. NCOA3 is a transcription cofactor with histone acetyltransferase^50^. NCOA3 is highly overexpressed in HCC and related to invasive phenotypes, although the roles of TM4SF5 in this regard have not been explored^60^. Furthermore, cytosolic NCOA3 associated with membranous TM4SF5 was stabilized via decreased ubiquitination upon ALB replenishment of SFM. Hepatocyte-specific PTEN deficiency results in steatohepatitis and HCC^61,62^. PTEN acetylation leads to inactivation of its lipid phosphatase activity^53^. Being consistent, we observed that cytosolic NCOA3 bound to PTEN, leading to its acetylation and inactivation and allowing high PIP_3_ levels in TM4SF5-positive hepatocytes. Thus, cytosolic NCOA3 may promote tumor progression by downregulating PTEN activity for PIP_3_-mediated macropinocytosis and thereby ALB uptake and energetics during cell migration. NCOA3 is enriched in the nucleus of HeLa cells via phosphorylation at Thr 24 (in addition to Ser 857 and Ser 860), which is supported by ERK1/2 activation upon EGF treatment^63^. We observed decreased pT^24^NCOA3 levels in the cytosol together with enhanced/sustained cytosolic NCOA3 levels, presumably leading to acetyltransferase activity toward its binder, PTEN. Thus, this study may provide further evidence of the role of cytosolic NCOA3 in HCC development and progression in a nutrient-restricted metabolic environment.

Our observations can indicate that hepatocyte TM4SF5 might support membrane ruffling or PIP_3_-mediated macropinocytosis depending on extracellular nutrient availability via cytosolic stabilization of NCOA3 and complex formation with TM4SF5 and PTEN, efficient ALB uptake and catabolism, ATP-linked respiration and cellular migration, presumably resulting in intrahepatic metastasis and multifocality. Although a lower extracellular ALB level is a well-known negative prognostic index of HCC, this study can suggest alternatively that a high [ALB]_serum_ (compared with those near lesions) might exaggerate liver malignancy via TM4SF5-dependent ALB uptake and bioenergetics, leading to tumor expansion.

## Supplementary information

Supplementary information

Uncut raw WB images.

Supplementary Movie 1.

Supplementary Movie 2.

Supplementary Movie 3.

Supplementary Movie 4.

## Data Availability

Data generated or analyzed during this study are included in this article. Further enquiries can be directed to the corresponding author.
