# Supplementary material for: Hepatocyte TM4SF5-mediated cytosolic NCOA3 stabilization and macropinocytosis support albumin uptake and bioenergetics for hepatocellular carcinoma progression
Source: Exp Mol Med. 2025 Apr 4;57(4):836–55. doi: 10.1038/s12276-025-01438-9 (PMC12046047; doi:10.1038/s12276-025-01438-9)
Supplement: Supplementary file 1 — Supplementary information [file 12276_2025_1438_MOESM1_ESM.pdf]

Hepatocyte TM4SF5-mediated cytosolic NCOA3 stabilization and macropinocytosis support albumin uptake and bioenergetics for hepatocellular carcinoma progression

Haesong Lee[1], Ji Eon Kim[1,2], Eun-Ae Shin[1], Yangie Pinanga[1], Kyung-hee Pyo[1], Eun Hae Lee[1], Wonsik Kim[1], Soyeon Kim[1], Chang Sup Lim[3], Kyung Chul Yoon[3], and Jung Weon Lee[1,2,*]

[1]Department of Pharmacy, College of Pharmacy, Seoul National University, Seoul 08826, Korea (Republic of)

[2]Research Institute of Pharmaceutical Sciences, College of Pharmacy, Seoul National University, Seoul 08826, Korea (Republic of)

[3]Department of Surgery, Seoul National University Boramae Medical Center, Seoul, 08826, Korea (Republic of)

Short Title: TM4SF5-NCOA3 mediated albumin uptake

Corresponding Author: Jung Weon Lee, jwl@snu.ac.kr

Supplementary Methods

Live imaging: Cells were transfected with pEGFP-AKT-PH (gifted from Professor Choi SY, University of Nebraska Medical Center, USA) for 48 h, and 10,000 cells were plated on an eight-well chamber slide (C7182, Nunc, Lab-TekII) precoated with collagen I (10 μM) and incubated in a humidified incubator at 37°C with 5% CO_2. Cells were serum-starved for 3 h and then did or did not undergo nutrient replenishment to SFM with GLU alone, ALB alone, or GLU+ALB. In a chamber maintained at 37ºC and 5% CO_2, cells were imaged every 15 s for 20 min before and immediately after ALB (3.6 mg/ml BSA) or GLU (25 mM) replenishment using a Nikon Eclipse Ti microscope with a C2 confocal system (Nikon). SNU761 cells transfected with $TM4SF5_{WT}$-mCherry for 48 h were live-imaged with an LSM880 laser scanning microscope with Airyscan (Carl Zeiss, Oberkochen, Germany) in a 37°C, 5% CO_2, and 95% humidity environment. Images were randomly captured and analyzed using ZEN imaging software (Carl Zeiss). Live images for multiple cells were recorded and representatively arranged for snapshots.

APEX2 staining and transmission electron microscopy (TEM): Cells were transfected with APEX2-TM4SF5 cDNA for 48 h and then manipulated for TEM imaging as previously described[1], using a FEI-Tecnai G2 Spirit Bio Twin TEM instrument. Images were taken using a 120-kV transmission electron microscope (Talos L120C, FEI, Hillsboro, OR).

Analysis of protein binding to TM4SF5 or ALB: TM4SF5-binding proteins were analyzed via proteomic analysis as previously described[2]. Briefly, SNU761 hepatic carcinoma cells were transfected with control or Strep-tag-conjugated $TM4SF5_{WT}$ plasmid for 48 h and then harvested using 1% Brij58 lysis buffer. Lysates were processed to pulldowns using streptavidin agarose (Thermo Fisher Scientific) before loading the pulldown to an SDS-PAGE gel. After in-gel trypsin digestion of proteins, peptides were analyzed by LC-MS/MS. The amino acid sequence was deduced after searching NCBI data using the SEQUEST program. Data were organized using the Human IPI database (Ver. 3.82) and analyzed by SCAFFOLD 3 analysis, with experiments repeated twice. Proteins binding to ALB were identified (https://thebiogrid.org/106715/summary/homo-sapiens/alb.html) from a previous study[3]. Proteins binding on both TM4SF5 and ALB were found to include NCOA3 and other proteins.

Immunohistochemistry: Human liver tumor and non-tumor samples were analyzed using hematoxylin and eosin staining or immunohistochemistry with anti-$TM4SF5_{EC2}$ (1:500), anti-NCOA3 (1:200), anti-PIP_3 (1:100, Z-P345, Echelon Biosciences Inc.), or anti-ALB (1:200, 213-MSM4-P1, Thermo Fisher Scientific) antibodies in blocking solution (10% normal goat serum at PBS) overnight at 4°C. Immunostaining was detected using a VECTASTAIN® ABC-HRP kit (Vector Laboratories, Burlingame, CA). Mayer's hematoxylin (Sigma-Aldrich) was used to counterstain nuclei. Ten random images per slide were obtained using a digital slide scanner (MoticEasyScan, Motic, British Columbia, Canada).

Live 3-dimensional (3D) holotomography: Cells were transfected with siRNA against non-specific sequence (siNS) or NCOA3 (siNCOA3, Table 1) using RNAiMAX (Invitrogen). Transfected cells (1.5×10^5/well) were seeded onto a confocal 35-mm clear cover glass-bottomed petri dish (#100350, SPL, Pocheon-si, South Korea) precoated with rat tail collagen I (#5056, Advanced Biomatrix) and incubated at 37ºC for 1 h. The dish was washed three times with PBS before cell seeding and then incubated at 37˚C for 15 h. BSA (3.6 mg/ml, Sigma) was added 30 min before time-lapse image captures (every 20 s) using a holotomography

microscope with laser interferometry to measure the 3D refractive index distribution at cellular edges while ruffling (Tomocube, HT-X1) at 37ºC within a 5% CO_2 humidified system (Tokai Hit, STXG-WELSX-SET) for 12 min. Captured images were visualized using TomoStudio X (Tomocube, Daejeon, South Korea).

PTEN phosphatase assay: PTEN proteins in whole-cell lysates prepared under different conditions were immunoprecipitated with anti-PTEN antibody, and their phosphatase activity was measured by ELISA according to the manufacturer's instructions (Echelon Biosciences Inc., Salt Lake City, UT).

1. Jung JW, *et al.* Liver-originated small extracellular vesicles with TM4SF5 target brown adipose tissue for homeostatic glucose clearance. *J Extracell Vesicles* **11**, e12262 (2022).
2. Kim HJ, *et al.* Dynamic and coordinated single-molecular interactions at TM4SF5-enriched microdomains guide invasive behaviors in 2- and 3-dimensional environments. *FASEB J* **31**, 1461-1481 (2017).
3. Zhou M, *et al.* An investigation into the human serum "interactome". *Electrophoresis* **25**, 1289-1298 (2004).
4. Tang Z, Kang B, Li C, Chen T, Zhang Z. GEPIA2: an enhanced web server for large-scale expression profiling and interactive analysis. *Nucleic Acids Res* **47**, W556-w560 (2019).

Supplementary Figure 1

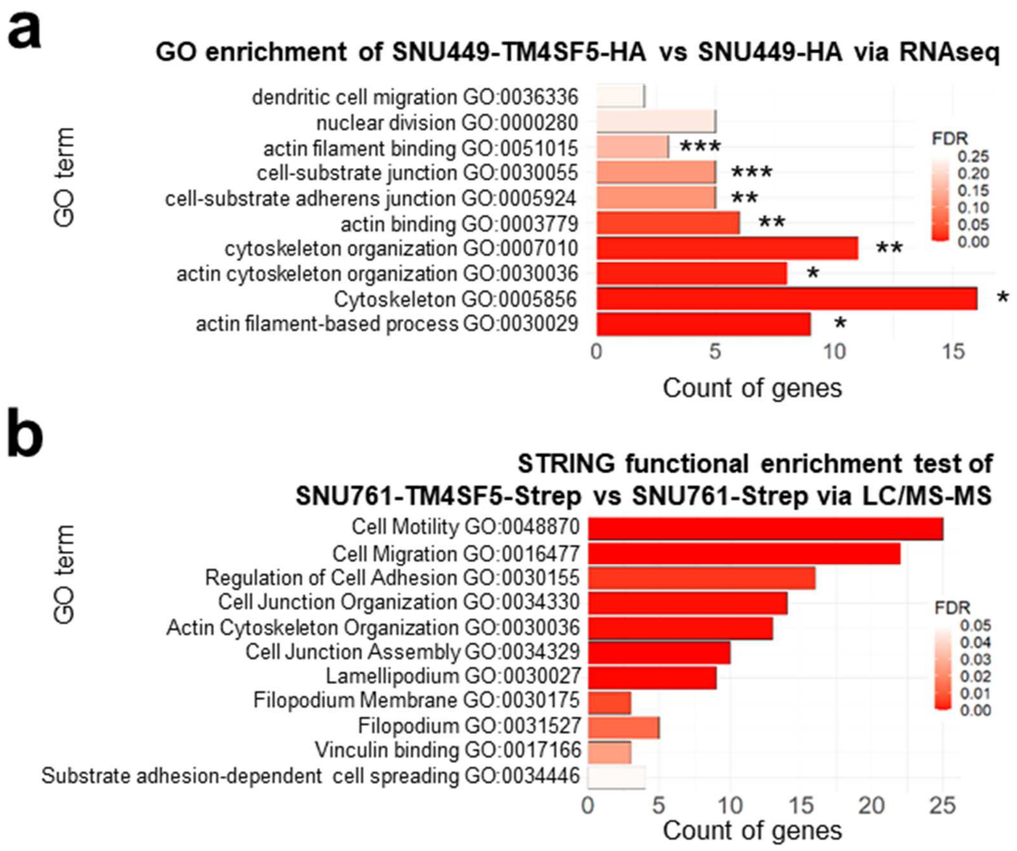

Supplementary Fig. 1. Gene ontology (GO) enrichment analysis and STRING functional enrichment analysis showing actin dynamics-relevant cellular functions in TM4SF5-overexpressing hepatocytes. (a) GO enrichment anlaysis of the RNA-Seq dataset (SRA accession number: PRJNA770813) revealed relavances of actin dynamic and cell migration to TM4SF5 expression in hepatocytes. (b) TM4SF5-binders identified by a proteomic analysis of TM4SF5-strep pulldowns using LC-MS/MS approaches[2], showed relevaces to actin organization and cell motility in STRING functional enrichment test. See also Fig. 1.

Supplementary Figure 2

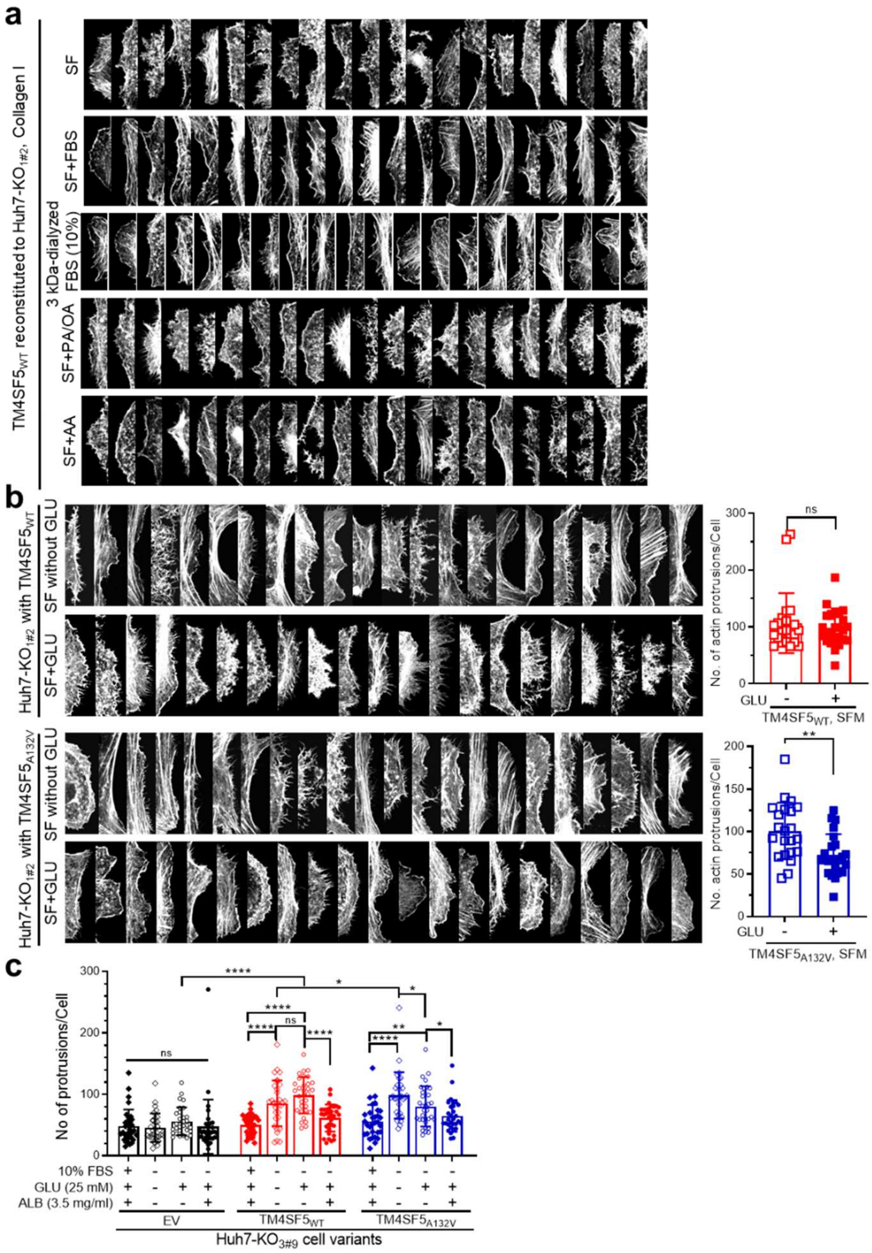

Supplementary Fig. 2. TM4SF5 expression in hepatocytes supported ALB- and/or GLU-mediated protrusive processes. (a-c) TM4SF5$_{WT}$ was transfected to Huh7-KO$_{1\#2}$ cells and then replated on collagen I-precoated slide glasses for 3 h. (a) Cells were then incubated with SFM with or without 10% FBS, 3 kDa-dilayzed FBS, palmitic acid and oleic acid (PA+OA, each 150 μM), or amino acid mixture (3 mg/ml) for 15 h before actin imaging. (b) Cells were incubated in GLU-depleted SFM or basal GLU-containing SFM for 15 h before actin imaging. Each image is from a different cell. (c) Control empty vector (EV), TM4SF5$_{WT}$, or TM4SF5$_{A132V}$ were transfected into Huh7-KO$_{3\#9}$ cell clone. Cells were then serum-starved and replenished with 10% FBS, GLU, or ALB for 15 h before actin imaging. Protrusive processes were quantified from random images of multiple cells using the Fiji plugin ADAPT (v1.193) for graphic presentations at mean±SD values. One dot depicts the number of protrusions per cell (i.e., all around a cell over edges), although the images in a and b show a part of the edge. *p*-values were calculated by unpaired Student's *t* tests or one-way ANOVA using Prizm software. Data represent three independent experiments. See also Fig. 1.

Supplementary Figure 3

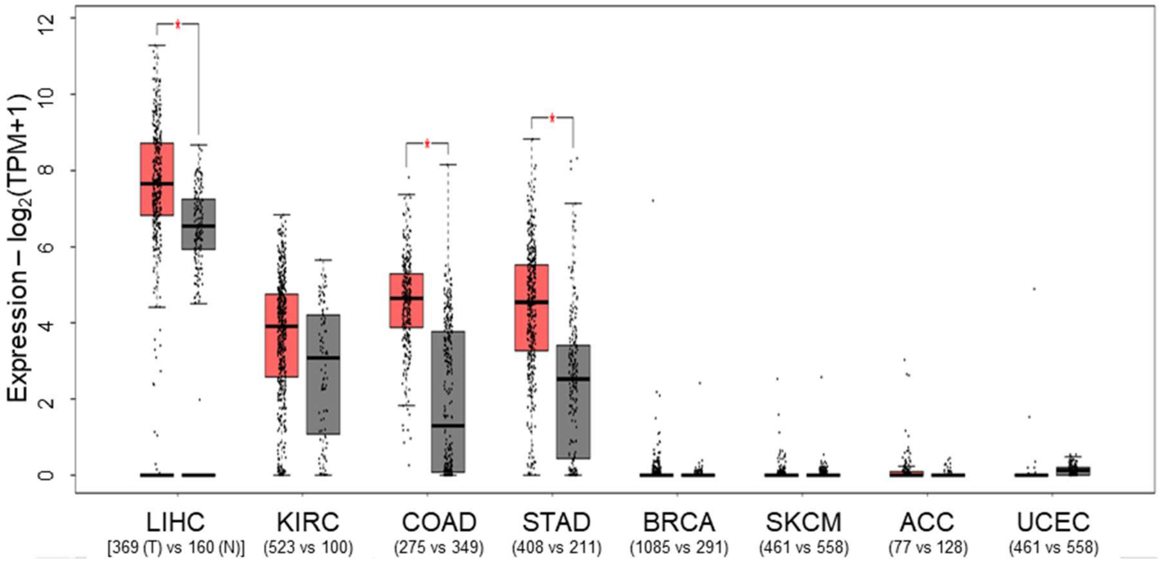

Supplementary Fig. 3. TM4SF5 expression in various tumor types of TCGA dataset. Various tumor types from public TCGA datasets combined with the GTXe projects[4] were analyzed for TM4SF5 expression between tumor and non-tumor samples. LIHC, liver hepatocellular *carcinoma; KIRC,* kidney renal clear cell *carcinoma; COAD,* colon adenocarcinoma; STAD, stomach adenocarcinoma; BRCA, breast invasive carcinoma; SKCM, skin cutaneous melanoma; ACC, adrenocortical carcinoma; UCEC, uterine corpus endometrial carcinoma. See also Fig. 2.

Supplementary Figure 4

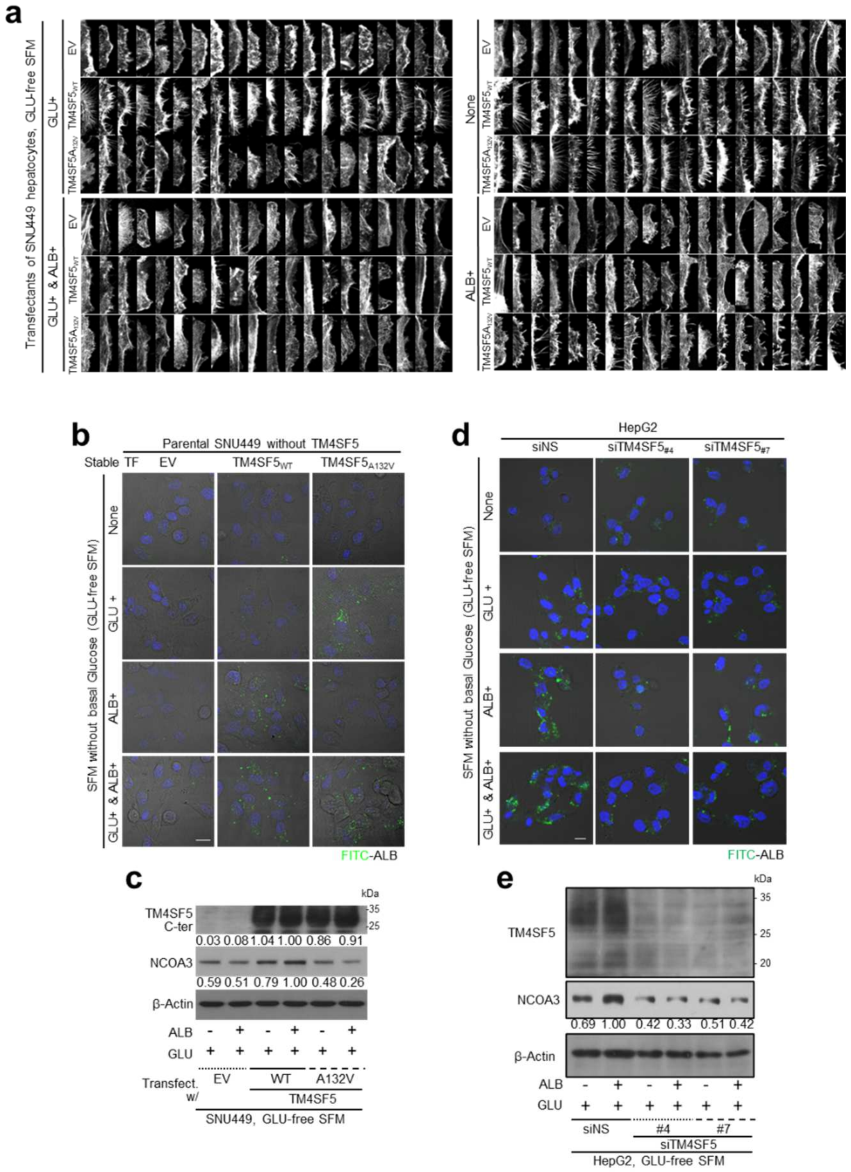

Supplementary Fig. 4. TM4SF5-mediated FITC-ALB uptake in SNU449 or HepG2 cells, depending on extracellular albumin and NCOA3 stabilization. Human hepatocyte cell lines, TM4SF5-lacking SNU449 (a-c) or endogenously TM4SF5-expressing HepG2 (d and e) cells were used for the cell variants with control vector transfection or TM4SF5-overexpression (a-c) or TM4SF5-suppression (d and e). The cells were serum-starved and lacked GLU for 3 hrs. Then the cells were either treated without (None) or with nutrient replenishment, as explained in the Materials and Methods. Replenishments of nutrient were done with None (i.e., GLU-free and ALB-free SFM), GLU alone (GLU+), ALB alone (ALB+) or GLU+ALB (GLU+ & ALB+) to GLU-free SFM for 15 h, before analysis. Analyses were done for morphological processes at the cellular edges (a), FITC-ALB uptake (b and d), and western blots for NCOA3 protein levels (c and e). During nutrient replenishment, FITC-ALB (0.01 mg/ml) was administrated before counting of macropinosomes within FITC-ALB-positive cells via analyzing multiple cells using a confocal microscope (b and d). Whole cell lysates were also prepared, before standard immunoblots. Ratio values of band intensities of certain immunoblot measured by Image J software were normalized to those of loading control (c and e). Data represent three independent experiments. See also Figs. 1 and 2.

Supplementary Figure 5

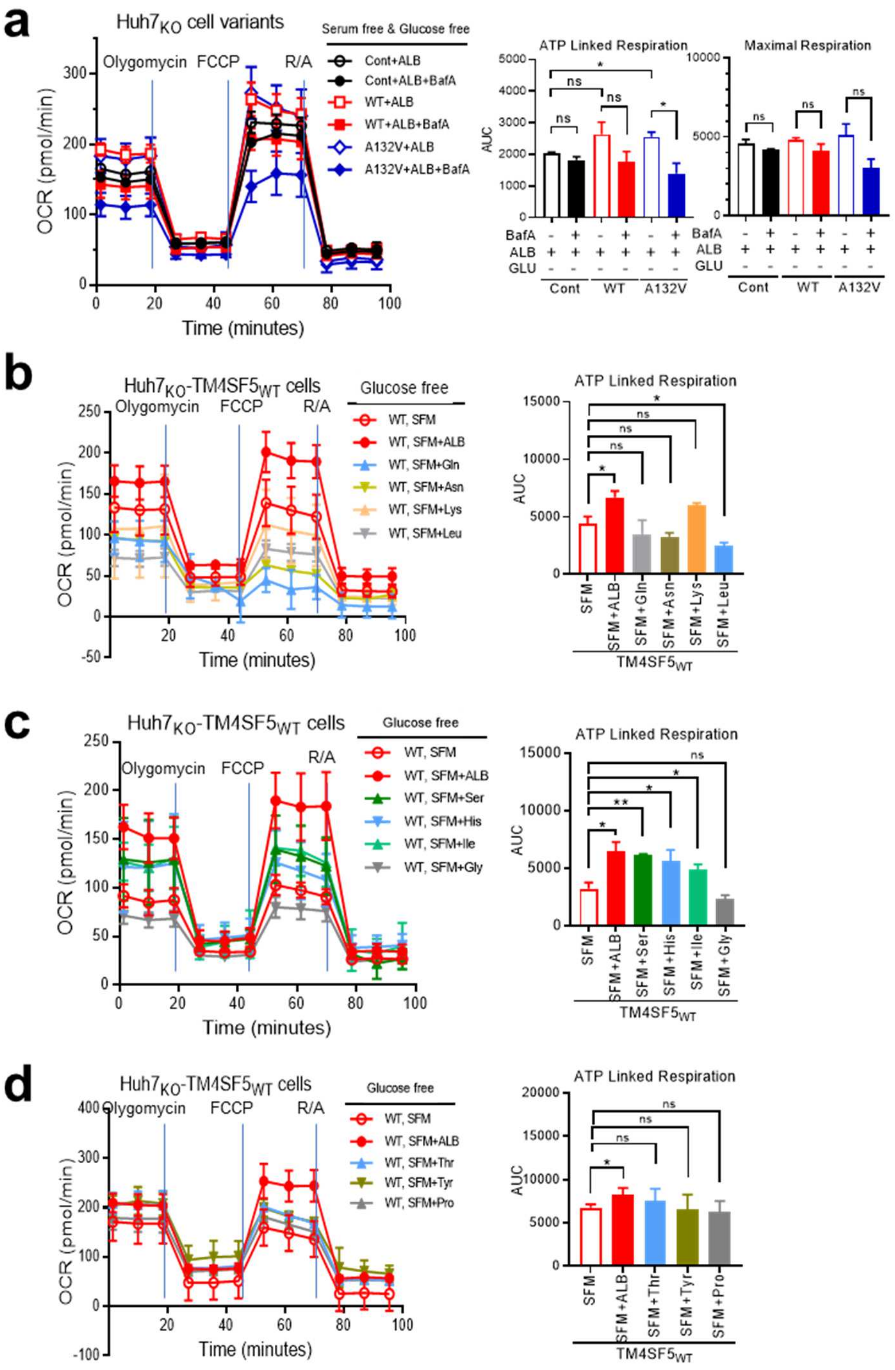

Supplementary Fig. 5. Mitochondrial OCR analysis upon H^+-ATPase inhibition or amino acid replenishment to SFM. (a) Huh7-$KO_{1\#2}$ cells transfected with control EV or $TM4SF5_{WT}$ cDNA were processed for serum and nutrient replenishment with or without bafilomycin A1 (BafA, a specific vacuolar-type H^+-ATPase inhibitor) before OCR measurement and AUC calculation of ATP-linked or maximal respiration. **(b-d)** Cells as in (a) were replenished with ALB or single amino acids before OCR measurement. ATP-linked respiration was calculated. * depicts $p<0.05$, and p-values were calculated by Student's t tests or one-way ANOVA. ns, non-significant. Data represent three independent experiments. See also Fig. 4.

Supplementary Figure 6

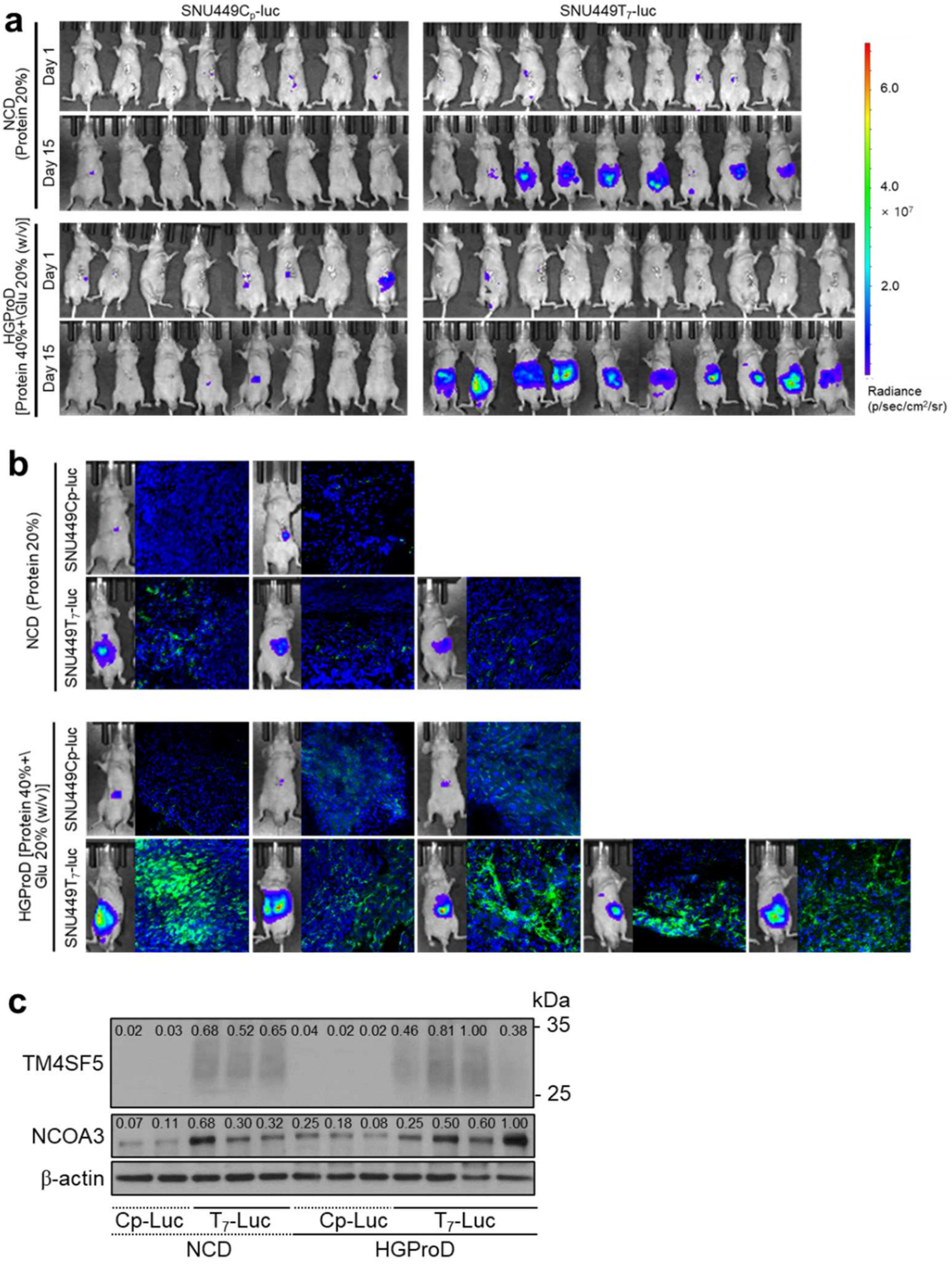

Supplementary Fig. 6. *In vivo* mouse model with liver-orthotopic injection of TM4SF5-luciferase hepatocytes for ALB uptake and intrahepatic metastasis. (a-c) SNU449C$_p$-luciferase (luc) or SNU449T$_7$-luc cells were orthotopically injected into the livers of 6-week-old BALB/cAnN-nude male mice as explained in Fig. 5g. Animals (n>8 per condition) were then fed a NCD with 20% protein or a HGProD with 20% GLU (w/v) and 40% protein until day 20. On day 1 or 15, *in vivo* imaging was performed for luciferase signal (a). On day 20, *in vivo* macropinocytosis-mediated FITC-ALB (that was intratumorally injected) uptake was analyzed. (c) The liver tissues from the animals were harvested for immunoblots against TM4SF5, NCOA3, and loading control. Ratio values of band intensities of certain immunoblots measured by using Image J software were normalized to those of loading control. See also Fig. 5.

Supplementary Figure 7

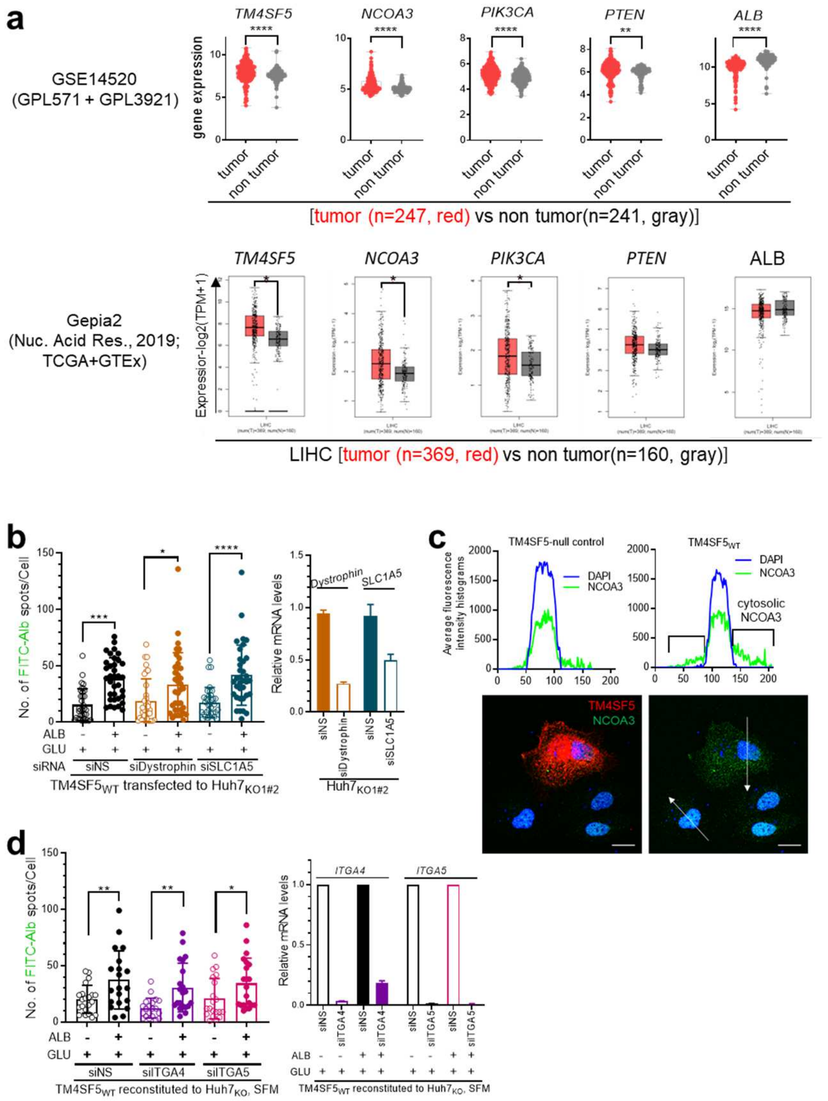

Supplementary Fig. 7. Analyses of molecules possibly related to macropinocytosis of extracellular ALB to hepatocytes. (a) Expression profiles of *TM4SF5*, *NCOA3*, *PI3KA*, *PTEN*, and *ALB* mRNAs from TCGA-LIHC combined with GTXe projects[4] or GSE14520 datasets. **(b)** Huh7-KO$_{1\#2}$ cells transfected with TM4SF5$_{WT}$ were transiently transfected with siRNA against control sequence (siNS), dystrophin (DMD, siDMD), or sodium-dependent neutral amino acid transporter (SLC1A5, siSLC1A5) for 24 h. One set of cells was processed for qRT-PCR for the gene (right graph). The other set of cells was serum-starved and then replenished with ALB to GLU+SFM for 15 h, before uptake analysis of FITC-ALB added to the media. **(c)** TM4SF5$_{WT}$ cells were immunostained for TM4SF5 (red) and NCOA3 (green) in addition to nuclear DAPI staining. Fluorescence intensities of TM4SF5$_{WT}$-positive or -negative or null (Control) cells were quantified over the arrows for graphic presentation. **(d)** Huh7$_{KO}$ cell variants were manipulated as in (b) except for transfection of siRNAs against *ITGA4* or *ITGA5*. mRNA expression of each gene was quantified via qRT-PCR (right), and *in vitro* macropinocytosis-mediated FITC-ALB uptake was evaluated for graphic presentation (left). *, ***, or **** depict $p<0.05$, 0.001, or 0.0001, respectively. *p*-values were calculated by Student's *t* tests or one-way ANOVA. Data represent three independent experiments. See also Figs. 6 and 8.

Supplementary Figure 8

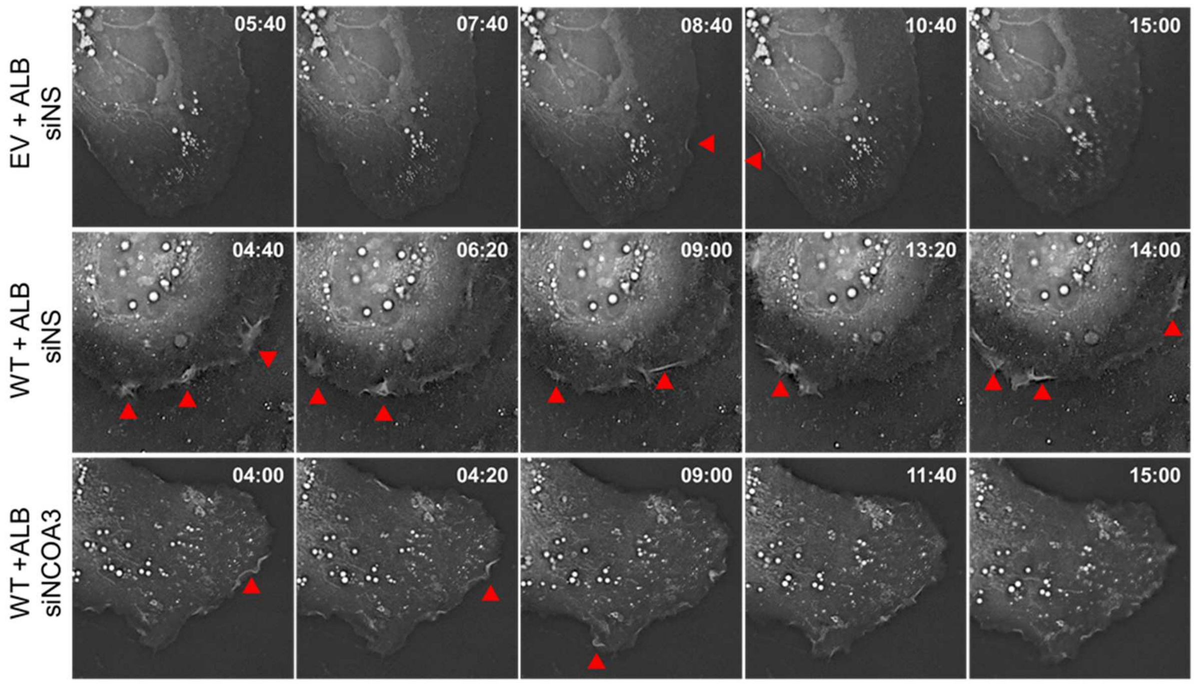

Supplementary Fig. 8. Membrane ruffling promoted by ALB replenishment to Huh7$_{KO}$-TM4SF5$_{WT}$ cells was abolished by NCOA3 suppression. Huh7$_{KO}$-cell variants transfected with empty vector (EV) or TM4SF5$_{WT}$ cDNA were transfected with siRNA against control no-specific sequence (siNS) or NCOA3 (siNCOA3) for 24 h. Cells were incubated for 15 h, and ALB was added to GLU+SFM 30 min before time-lapse imaging (for 15 min at 20-s intervals) using live 3D holotomography microscope. Red arrowheads indicate active ruffling. See also Fig. 6.

Supplementary movie spreadsheet

Supplementary Movie 1. TM4SF5-positive hepatocytes showed active membrane ruffling. SNU761 transfected with TM4SF5 WT-mCherry for 48 h was imaged by Airyscan higher-resolution microscopy. Time-lapse fluorescence microscopy videos were taken with an inverted, confocal, laserscanning microscope (LSM880 confocal microscope; Carl Zeiss) equipped with an Airyscan super-resolution imaging module and a363/1.40-NAPlan-Apochromat Oil Differential Interference Contrast M27 objective lens (Carl Zeiss). LSM observations were all performed at room temperature. mCherry was detected by excitation at 561 nm by using a Diode-Pumped Solid-State laser (0.3% laser output) with 570-nm long-pass filter. Microscopy images and videos were processed and analyzed by using Zen 2012 software. Videos were filmed at 8 frames/s. See also Figure 3a.

Supplementary Movie 2. TM4SF5-negative Huh7$_{KO}$ hepatocytes in serum free culture media showed less active membrane ruffling. Ten thousand Huh7$_{KO}$ cells (Control) were plated on cover glasses precoated with 10 μg/ml collagen I (354236, BD Biosciences, San Jose, CA) in PBS at room temperature (RT) for 1 h or an 8-well chamber slide (C7182, Nunc, Lab-TekII) and incubated in a humidified incubator at 37°C with 5% CO_2 for 16 h. The cells were then transfected with pEGFP-AKT-PH constructs using Lipofectamine 3000 for 24 h. Cells were then randomly visualized in serum-free media at a 37°C, 5% CO_2 chamber, using a Nikon Eclipse Ti microscope with a C2 confocal system. Images were analyzed using NIS-Elements software (Nikon, Melville, NY). See also Figure 3g.

Supplementary Movie 3. TM4SF5$_{WT}$-positive hepatocytes in serum free culture media showed active membrane ruffling. Ten thousand TM4SF5-positive hepatocytes (Huh7$_{KO}$ cells stably transfected with TM4SF5 WT construct) were plated on cover glasses pre-coated with 10 μg/ml collagen I (354236, BD Biosciences, San Jose, CA) in PBS at room temperature (RT) for 1 h or an 8-well chamber slide (C7182, Nunc, Lab-TekII) and incubated in a humidified incubator at 37°C with 5% CO_2 for 16 h. The cells were then transfected with pEGFP-AKT-PH constructs using Lipofectamine 3000 for 24 h. Cells were then randomly visualized in serum-free media at a 37°C, 5% CO_2 chamber, using a Nikon Eclipse Ti microscope with a C2 confocal system. Images were analyzed using NIS-Elements software (Nikon, Melville, NY). See also Figure 3g.

Supplementary Movie 4. TM4SF5$_{A132V}$-positive hepatocytes in serum free culture media showed active membrane ruffling. Ten thousand TM4SF5-positive hepatocytes (Huh7$_{KO}$ cells stably transfected with TM4SF5$_{A132V}$ construct) were plated on cover glasses pre-coated with 10 μg/ml collagen I (354236, BD Biosciences, San Jose, CA) in PBS at room temperature (RT) for 1 h or an 8-well chamber slide (C7182, Nunc, Lab-TekII) and incubated in a humidified incubator at 37°C with 5% CO_2 for 16 h. The cells were then transfected with pEGFP-AKT-PH constructs using Lipofectamine 3000 for 24 h. Cells were then randomly visualized in serum-free media at a 37°C, 5% CO_2 chamber, using a Nikon Eclipse Ti microscope with a C2 confocal system. Images were analyzed using NIS-Elements software (Nikon, Melville, NY). See also Figure 3g.
