# Supplementary figures and images for: Hepatocyte TM4SF5-mediated cytosolic NCOA3 stabilization and macropinocytosis support albumin uptake and bioenergetics for hepatocellular carcinoma progression
Source: Exp Mol Med. 2025 Apr 4;57(4):836–55. doi: 10.1038/s12276-025-01438-9 (PMC12046047; doi:10.1038/s12276-025-01438-9)

Fig. 1b

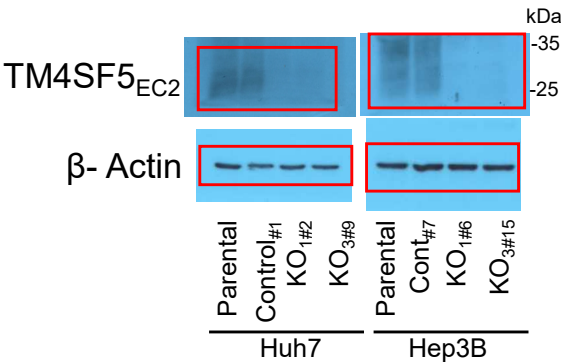

Fig. 1k

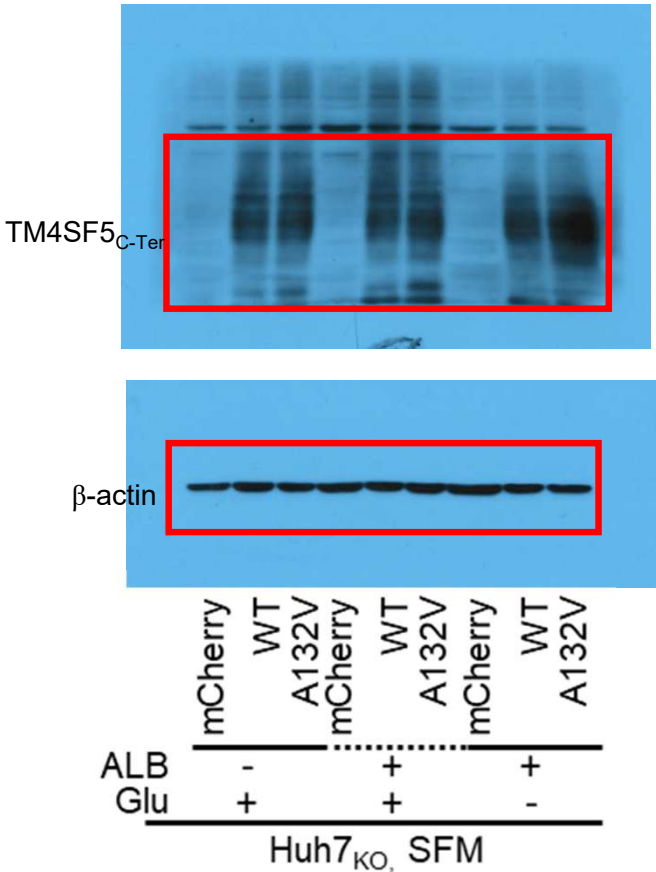

**Fig. 2g**

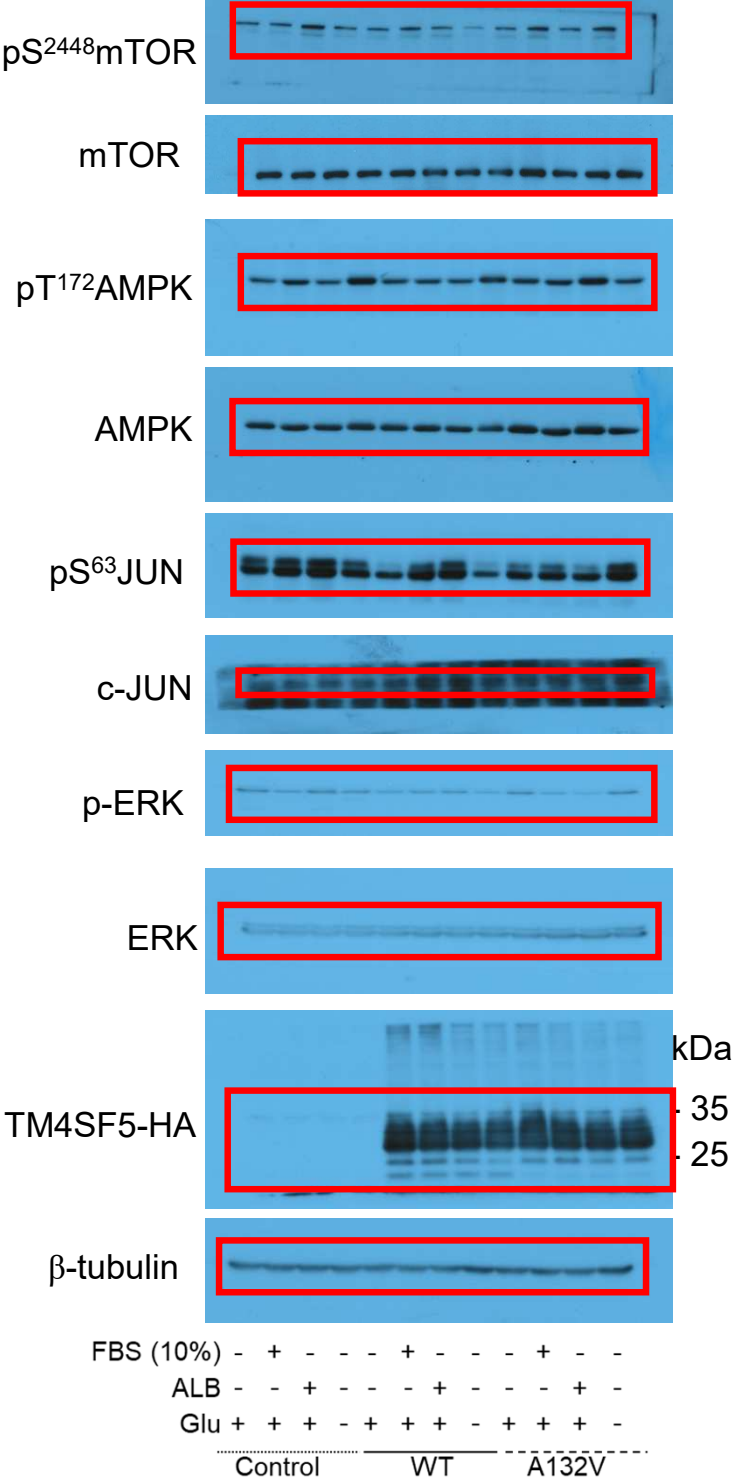

**Fig. 3d**

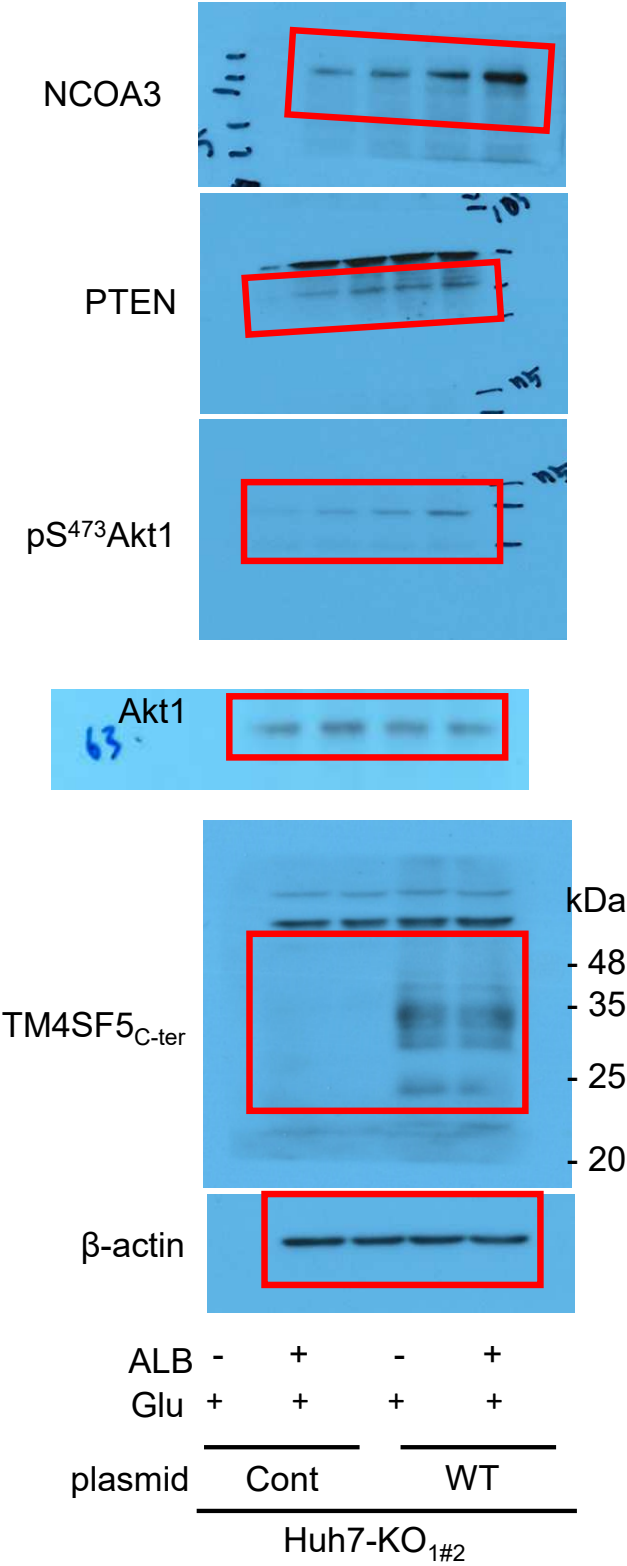

**Fig. 6l**

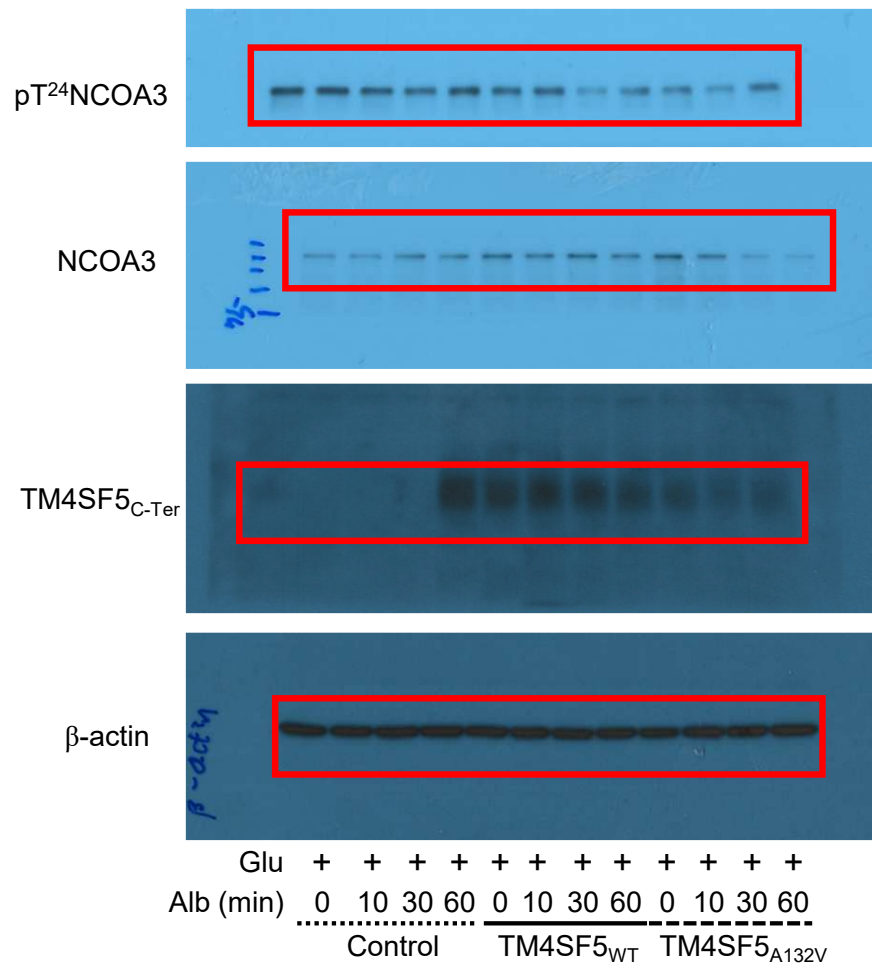

**Fig. 7a**

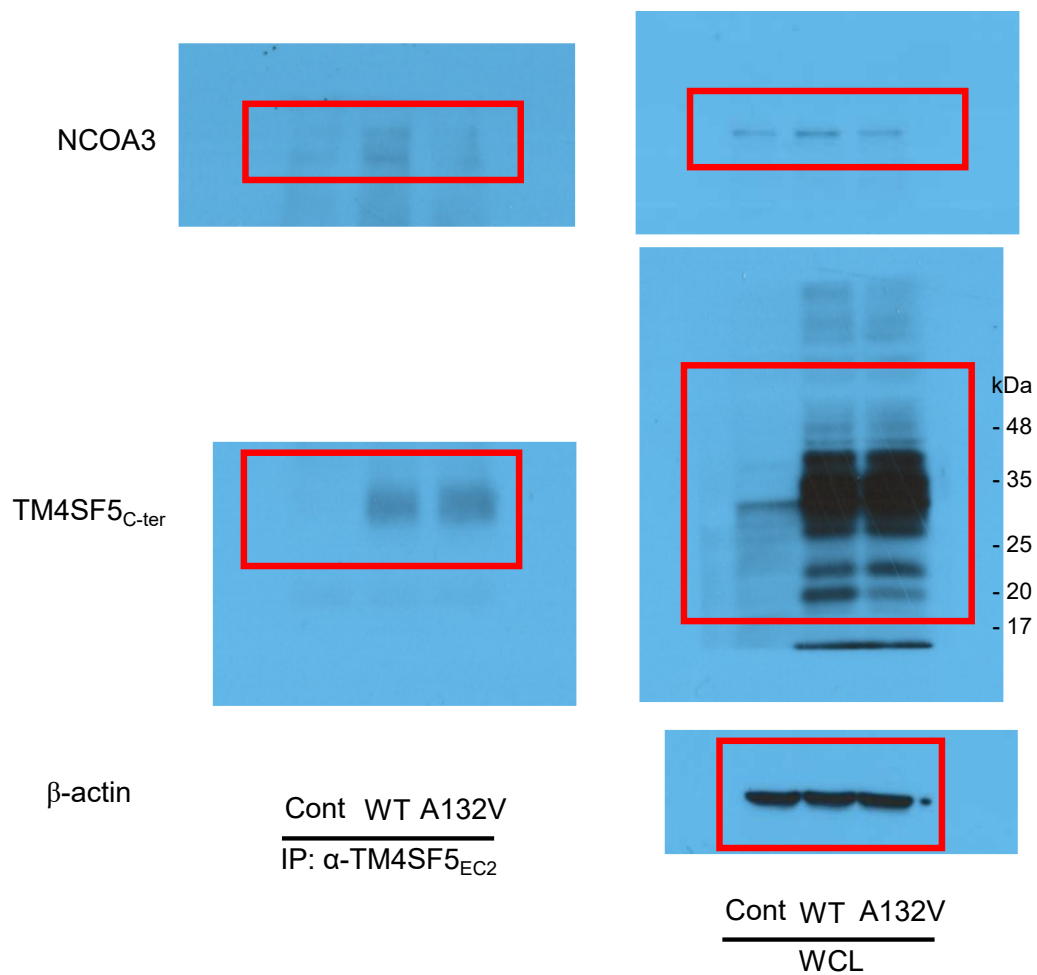

Fig. 7b

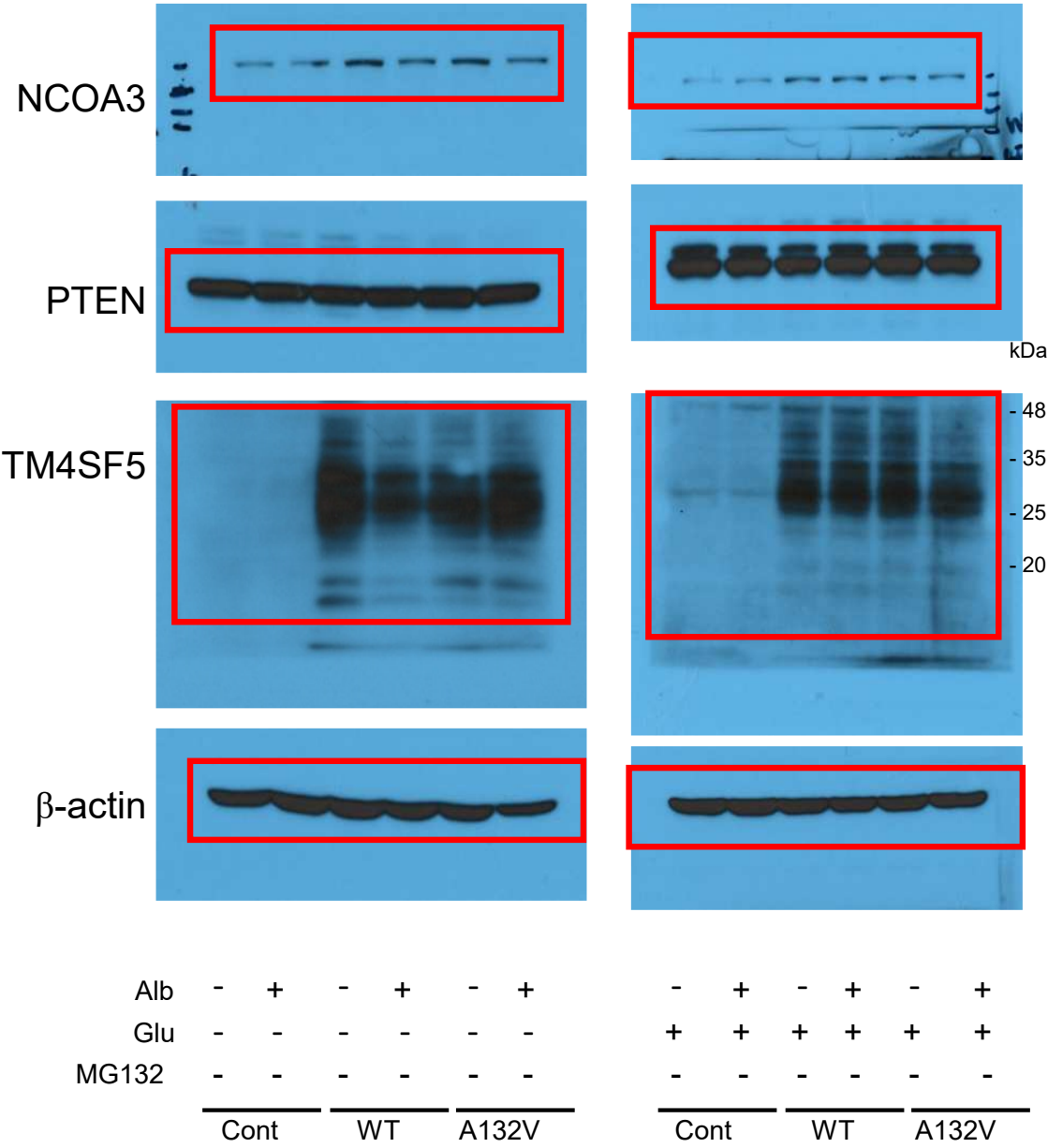

Fig. 7c

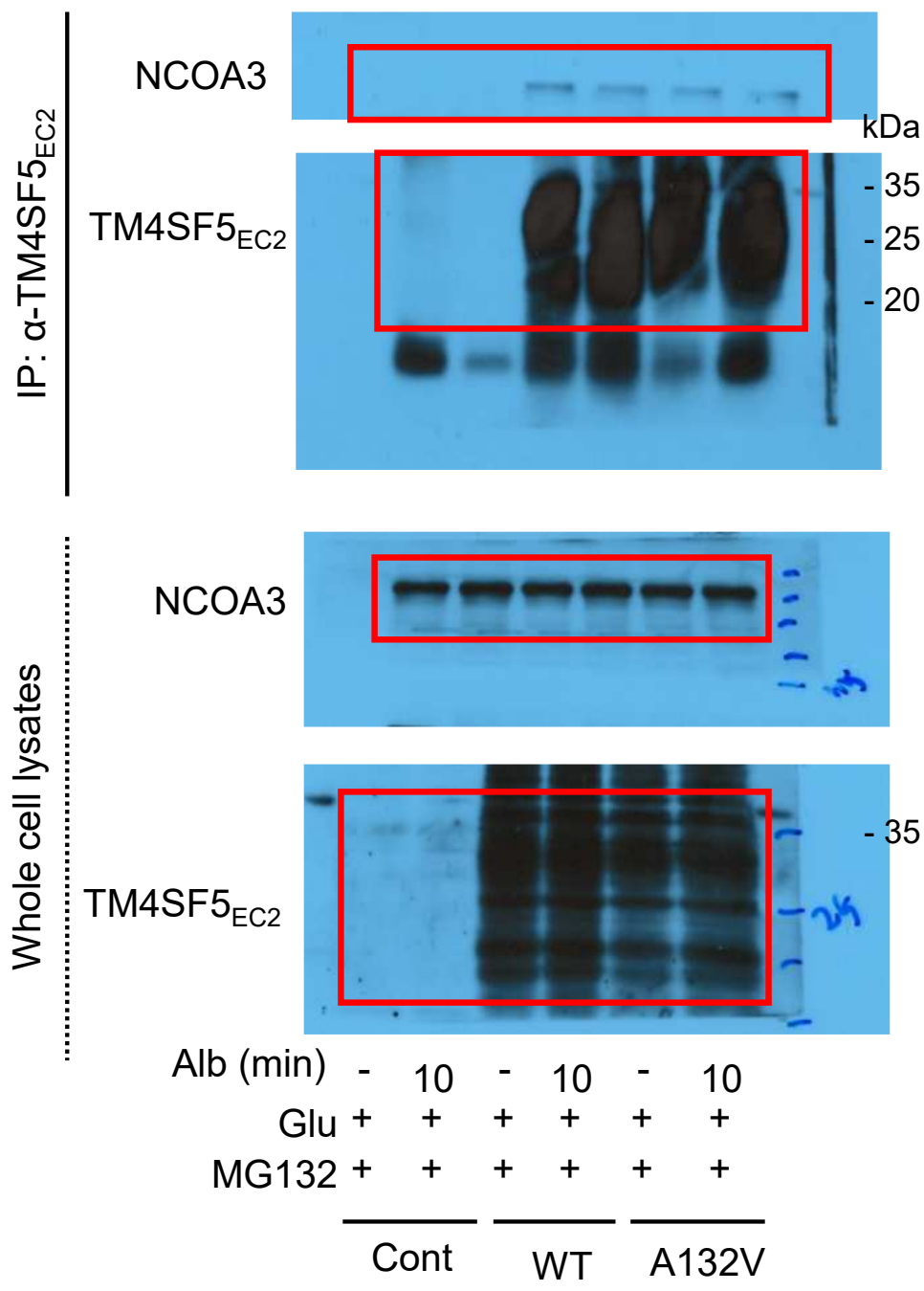

Fig. 7d

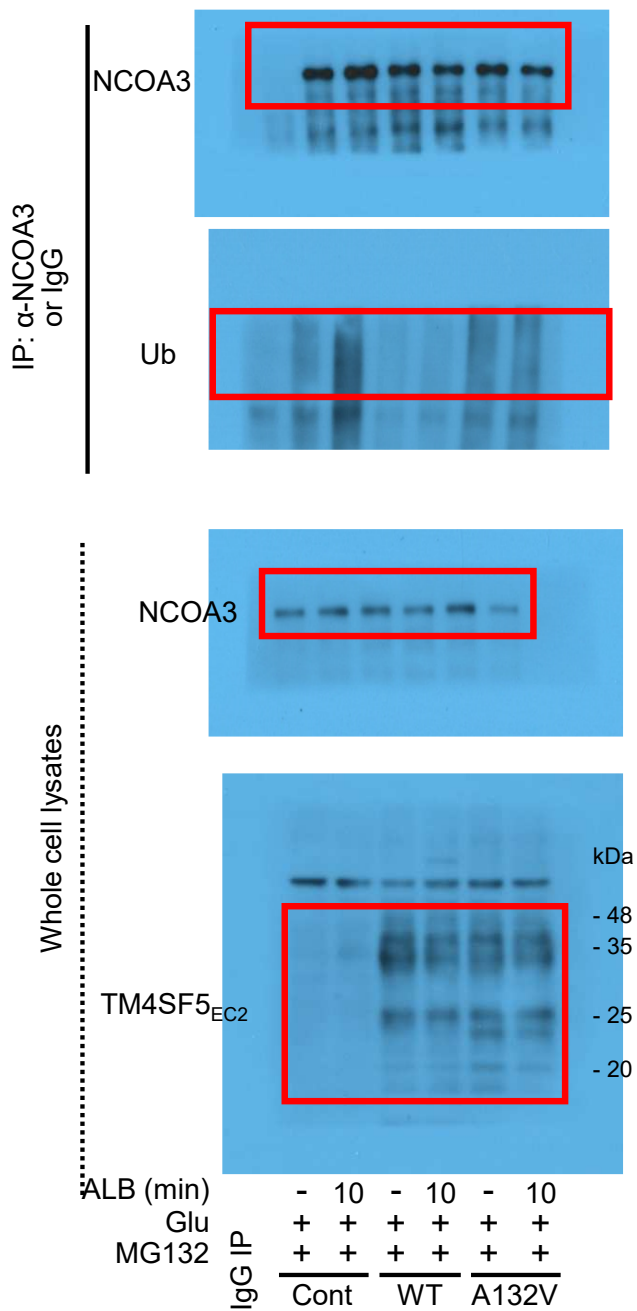

Fig. 7e

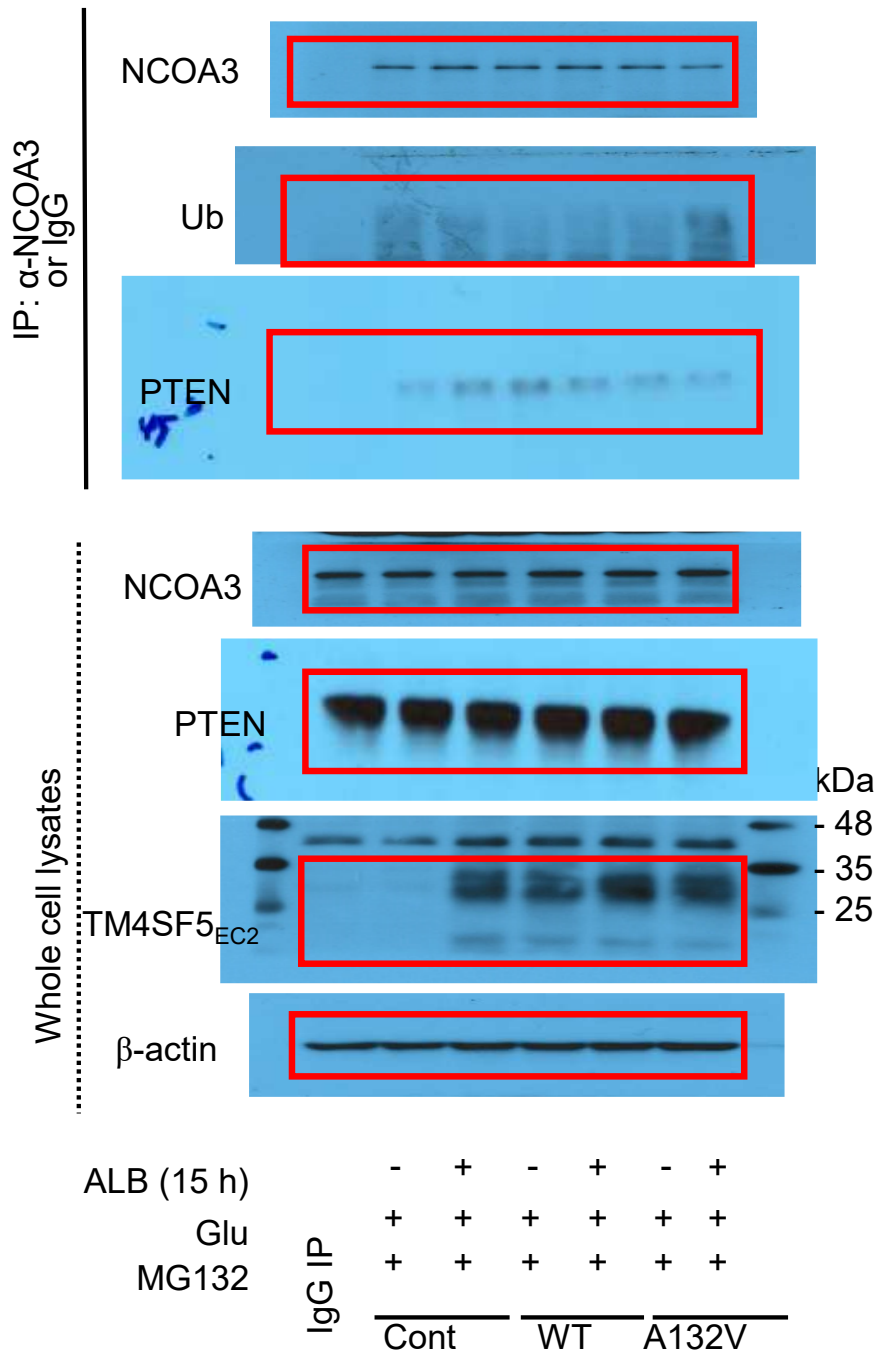

Fig. 7f

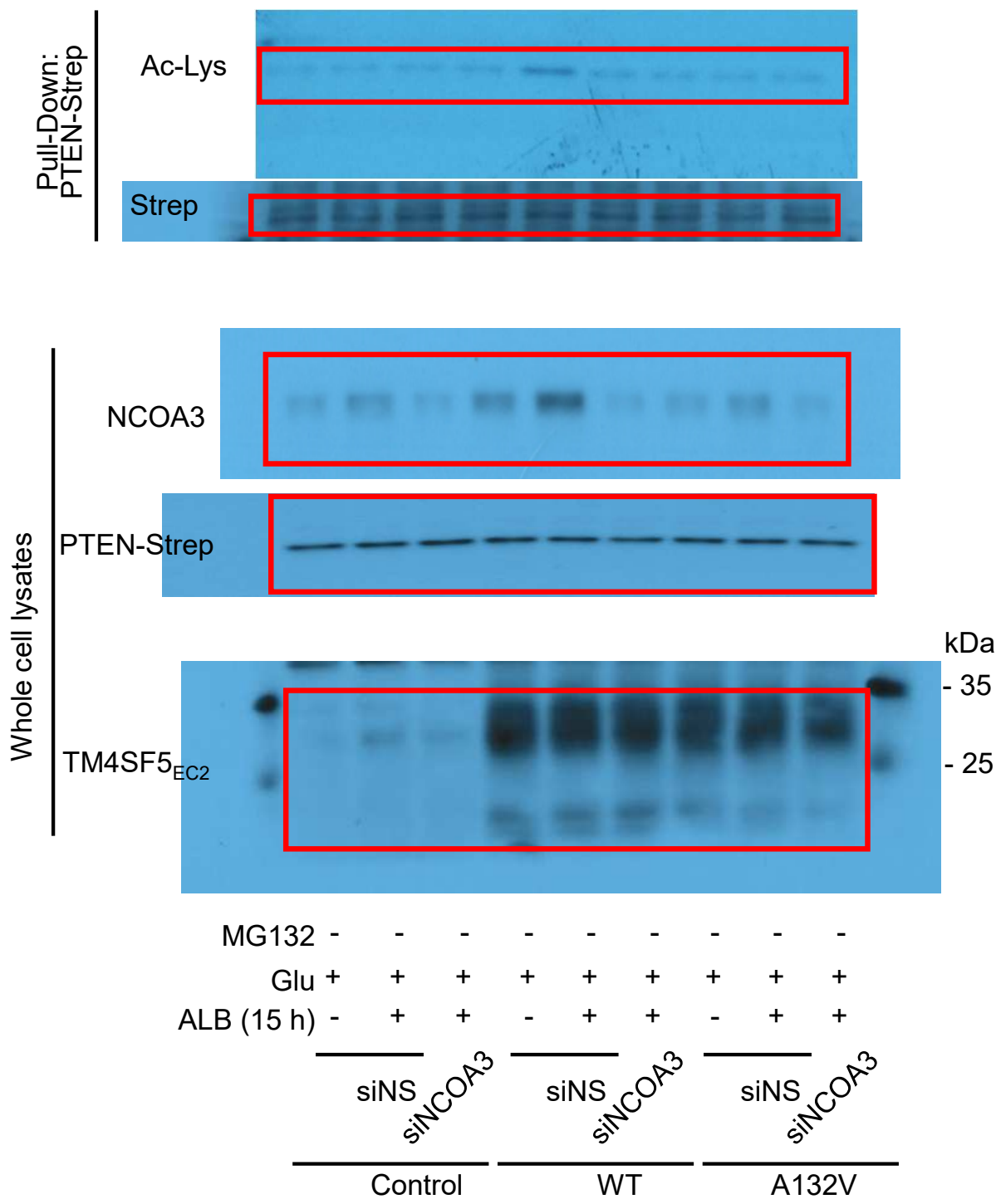

**Fig. 7g**

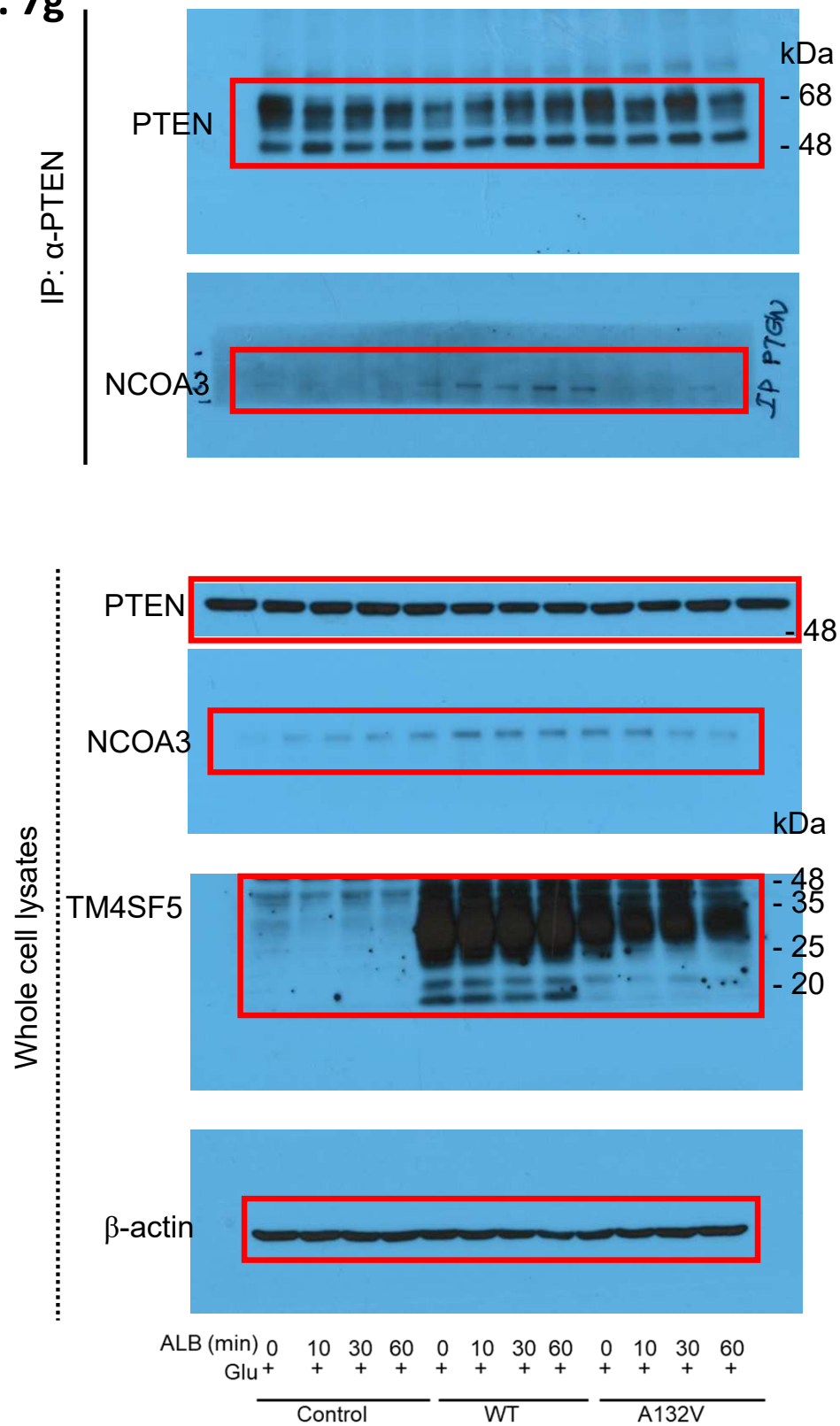

Fig. S4c

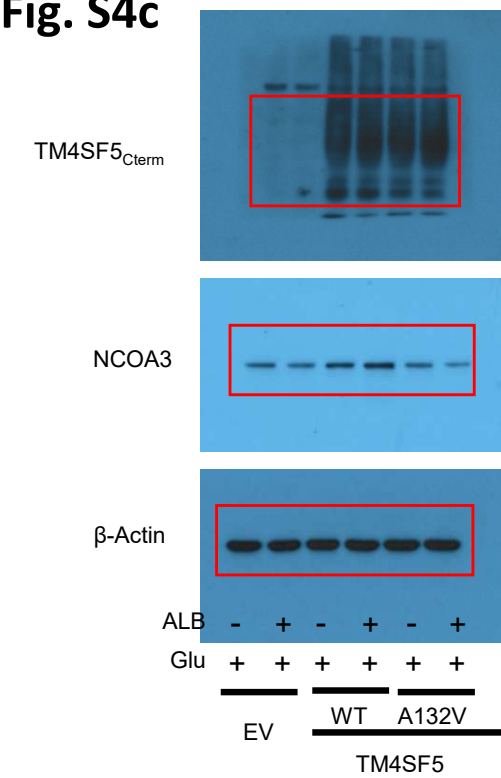

Fig. S4e

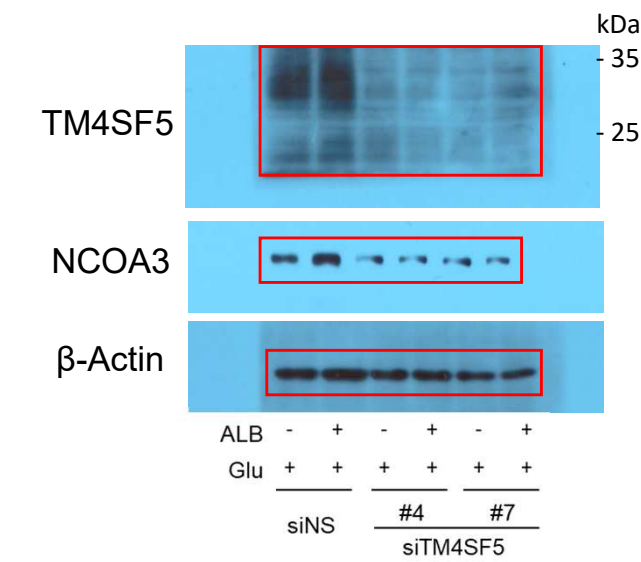

Fig. S6c

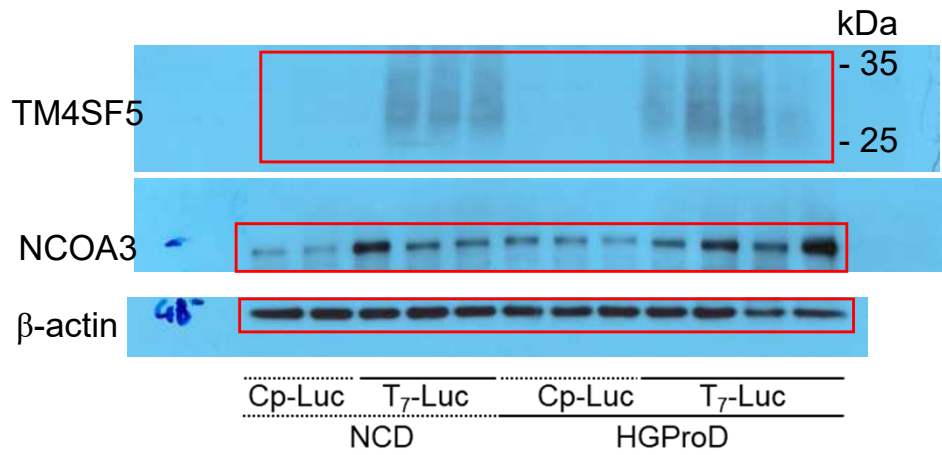

Supplement: Supplementary file 2 — Uncut raw WB images. [file 12276_2025_1438_MOESM2_ESM.pdf]
